# Dendropanax Morbiferus and Other Species from the Genus Dendropanax: Therapeutic Potential of Its Traditional Uses, Phytochemistry, and Pharmacology

**DOI:** 10.3390/antiox9100962

**Published:** 2020-10-08

**Authors:** Rengasamy Balakrishnan, Duk-Yeon Cho, In Su-Kim, Dong-Kug Choi

**Affiliations:** Department of Applied Life Sciences and Integrated Bioscience, Graduate School, Konkuk University, Chungju 27478, Korea; rmbalabio@gmail.com (R.B.); whejrdus10@kku.ac.kr (D.-Y.C.); kis5497@kku.ac.kr (I.S.-K.)

**Keywords:** *Dendropanax*, phytoconstituents, oxidative stress, antioxidant, neuroprotective, anti-inflammation

## Abstract

The *Dendropanax* genus is a kind of flowering plant in the family of Araliaceae that encompasses approximately 91 to 95 species. Several *Dendropanax* species are used as traditional medicinal plants, extensively used Korea and South America and other parts of the world. Almost every part of the plant, including the leaves, bark, roots, and stems, can be used as traditional medicine for the prevention and management of a broad spectrum of health disorders. This paper sought to summarizes the ethnopharmacological benefits, biological activities, and phytochemical investigations of plants from the genus *Dendropanax,* and perhaps to subsequently elucidate potential new perspectives for future pharmacological research to consider. Modern scientific literature suggests that plants of the *Dendropanax* genus, together with active compounds isolated from it, possess a wide range of therapeutic and pharmacological applications, including antifungal, anti-complement, antioxidant, antibacterial, insect antifeedant, cytotoxic, anti-inflammatory, neuroprotective, anti-diabetic, anti-cancer, and anti-hypouricemic properties. The botanical descriptions of approximately six to 10 species are provided by different scientific web sources. However, only six species, namely, *D. morbiferus, D. gonatopodus, D. dentiger*, *D. capillaris*, *D. chevalieri,* and *D. arboreus*, were included in the present investigation to undergo phytochemical evaluation, due to the unavailability of data for the remaining species. Among these plant species, a high concentration of variable bioactive ingredients was identified. In particular, *D. morbifera* is a traditional medicinal plant used for the multiple treatment purposes and management of several human diseases or health conditions. Previous experimental evidence supports that the *D. morbifera* species could be used to treat various inflammatory disorders, diarrhea, diabetes, cancer, and some microbial infections. It has recently been reported, by our group and other researchers, that *D. morbifera* possesses a neuroprotective and memory-enhancing agent. A total of 259 compounds have been identified among six species, with 78 sourced from five of these species reported to be bioactive. However, there is no up-to-date information concerning the *D. morbifera*, its different biological properties, or its prospective benefits in the enhancement of human health. In the present study, we set out to conduct a comprehensive analysis of the botany, traditional medicinal history, and medicinal resources of species of the *Dendropanax* genus. In addition, we explore several phytochemical constituents identified in different species of the *Dendropanax* genus and their biological properties. Finally, we offer comprehensive analysis findings of the phytochemistry, medicinal uses, pharmacological actions, and a toxicity and safety evaluation of the *D. morbifera* species and its main bioactive ingredients for future consideration.

## 1. Introduction

Natural products continue to make extensive contributions to human health, alongside modern medicine and in drug discovery. According to reports from the World Health Organization (WHO), universally, 80% of people still trust and use natural medicines; at present, several drugs also owe their inception to medicinal plants. The search for new molecules with therapeutic potential among natural resources is ongoing, and has resulted in numerous important findings, that include anti-oxidant, anti-inflammatory, analgesics, antibiotics, and anti-cancer components. The widespread genetic diversity of natural medicinal plants offers several important opportunities for the improvement of humankind like food production containing single ingredients with nutritional value and medicinal properties [1,2,3,4,5]. Currently, phytotherapies and pharmacologists represent an around $14 billion market per year, or approximately 5% of the current $280 billion per annum world market for traditional medicine. As a thing of noteworthy, there is a huge difference exists between developed and developing countries in making use of herbal products in their medications. Comparatively the developing countries use plant products in their 25% and 80% of medication strategies [6]. Of the 56% of currently recommended, or prescribed synthetic drugs linked to natural sources in some fashion, 24% are derived from plant species, nearly 9% are synthetic drugs developed using natural products as the reference, around 6% are isolated directly from natural plant species, and 5% are of animal source [7]. However, still there is a huge amount of drug resources being untapped. In the cradle of nature having around 350,000 to 550,000 plant species, of which less than 20% species have been explored for therapeutic applications [8]. The *Dendropanax* genus belongs to the family Araliaceae, and is found throughout the south-western region of East Asia, Korea, and Japan; furthermore, it is considered a neotropical genus containing approximately 91 to 95 species distributed from the Malay Peninsula and Central South America [9,10,11]. Indeed, all species but *D. morbiferus* are native to the tropics of South Korea, predominantly found on Jeju Island [12,13,14,15]. Most species of the genus *Dendropanax* is historically important trees that are largely evergreen, and numerous parts of these plant species commonly been used by indigenous peoples to recover from various disorders, atopic dermatitis, asthma, sore throat, anemia, anti-oxidative, viral infections and kidney problems [16,17,18,19]. The traditional use of *Dendropanax* species was used for treating inflammatory and arthritis conditions [9]. Elsewhere, the leaves, stems, roots, and seeds of some species have been used as a form of alternative medicine for lung problems, cough, and neurological disorders, including paralysis, stroke, and migraine [19,20].

Several other parts of the *D. morbiferus* plant including the edible leaf, bark, seeds, stems, and roots have been recognized as alternative, folkloric medicine and food additives and registered with Ministry of Food and Drug Safety, Korea Food and Drug Administration (foodsafetykorea.go.kr). Many scientific reports have previously highlighted the antioxidant, nephroprotective, antidiabetic, and anticarcinogenic activities of this plant species [21,22,23]. Recently, *D. morbiferus* leaves have also been reported to potentially improve hippocampal function and alleviated endogenous antioxidant levels in mercury-induced neurotoxic rats [24]. It has recently been determined, by both ourselves and other researchers, that the aqueous leaf extract of *D. morbiferus* has potentially restored behavioral deficits and significantly diminished neuro-inflammatory–mediated activation in microglia, induced in 1-methyl-4-phenyl-1, 2, 3, 6-tetrahydropyridine (MPTP) neurotoxic C57Bl/6N mice [13].

**Figure 1:**
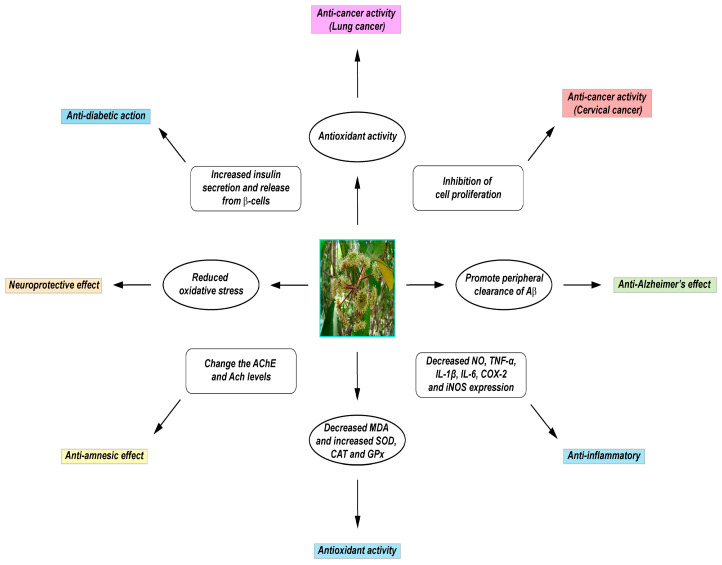
Pharmacological activities of *D. Morbiferus*.

Indeed, the traditional uses of *D. morbiferus* have been proved to date by recent pharmacological studies. Previous studies have determined that *D. morbiferus* performs various biological activities, including antioxidant [16,25], anti-inflammatory [26,27], anti-amnesic [14], neuroprotective [13,28], anti-cancer [29], anti-diabetic [30,31], hepatoprotective [32,33], immunomodulatory [27,34], antibacterial and antifungal [35], antiplasmodial [36], cytotoxic [37,38], and larvicidal [39] functions (Figure 1). Recent research has also demonstrated that *D. morbiferus* could be used as a prebiotic and probiotic agent against various pathogens [40]. In particular, the Korea Forest Research Institute, which investigated the biological activities of trees, declared that *D. morbiferus* contained protective components against dental caries.

In the present investigation, the potential of plants of the *Dendropanax* genus to yield several bioactive samples, both from pure compounds and crude extracts, may authenticate a degree of effectiveness concerning the impacts of their biological activities. Herein, a critical evaluation of the molecular basis of cellular and animal models of pharmacological activities, the mode of actions, toxicology and ethnomedicinal uses of the *D. morbiferus* species is presented. This study ultimately aims to offer a well-designed summary and possible recommendations for existing studies to consider that might direct future research. The total number of *Dendropanax* genus-related publications registered in Pub Med literature database is shown in (Figure S1).

## 2. Taxonomy, Distribution, and Description of *Dendropanax* Species

**Figure 2:**
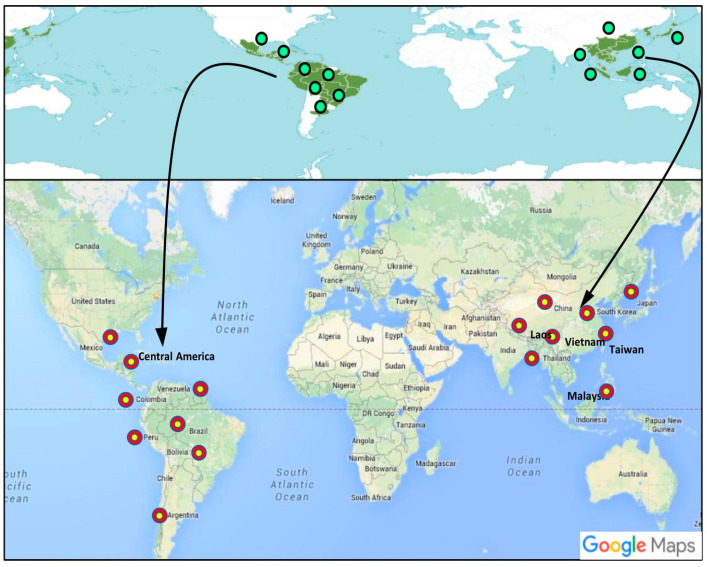
Global distribution (Green dots) map of *Dendropanax* showing the abundance in worldwide (http://www.ipni.org and http://apps.kew.org/wcsp/). The Google maps occurrence of the species within southwestern region countries is shown in red dots (https://images.app.goo.gl/wCJHWkM4fDJBFakr5).

Araliaceae is a broadly distributed family that presently consists of 55 genera, 1500 species, and one subfamily (Aralioideae) [41], which the genus *Dendropanax* belongs to. This genus of *Dendropanax* has the highest species diversity, and consists of 91 to 95 species that are distributed across China, Korea, Thailand, Malaysia, Vietnam, Japan, Taiwan, Laos, Mexico, Central America, Argentina, Colombia, Peru, Bolivia, and Venezuela, of which five are found in Brazil [42,43,44] (Figure 2). The *Dendropanax* genus presents a high level of morphological diversity. The species of the genus *Dendropanax* plants are evergreen trees occurring in tropical and subtropical regions, which is a small understory shrub. In these plants, the stem is vertical; can grow to a height of 5 m; and may be unlobed, or have either two or three to five lobes. Plants of the *Dendropanax* genus grow moderately, at a rate of up to 1.5 feet per year [45]. The flowers are yellowish-green, and their smooth bark is pale yellowish-gray, borne on umbels, up to 0.4 wide. The species of the genus *Dendropanax* prefer to grow in partial shade on well-drained soil. *Dendropanax* species generally have a solitary terminal flower clusters and inflorescence morphology, and also there is the presence of simple umbel to a small compound inflorescence within the same species been observed in *D. chevalieri* and *D. dentiger* [12,46]. Species of *Dendropanax* is limited in its distribution and have limited genetic variation out of genetic disorders or close mating, compared to the other species of trees [14]. Among the species of the genus *Dendropanax*, the scientific name of the Hwangchil tree is *D. morbifera,* and this plant is widespread in South-western Brazil, Korea, and Japan. In Greek, “dendro” means tree, and “panax” means panacea [19]. *D. morbifera* is a golden color medium-sized tree of 15 meters in height with oval-shaped duck feet like leaves. Blooming of *D. morbifera* flowers happens between June to August and produce black fruits between the September and November [12]. The *D. morbifera* tree grows mainly in wet tropical regions of warm temperate places around the world, especially in Korea [15]. 

## 3. Phytochemistry of *Dendropanax* Species

Despite the growing interest in pharmacologically active phytochemicals sourced *Dendropanax* species, as far as we know, only a few studies considering the phytochemical and pharmacological features of this genus plant species have been conducted to date. The *Dendropanax* genus has been shown to contain polyphenols, flavonoids, tannins, pyrimidines, essential oils, terpenoids, phenol carboxylic acids, and alkaloids. From the available literature, the species *D. morbifera* has been the most-widely investigated, although *D. dentiger, D. arboreus, D. chevalieri, D. proteus,* and *D. querceti* have also been examined. In recent decades, the phytochemical and main bioactive compounds of *D. morbifera* [47,48,49,50,51,52] (Table 1 and Table 2) and other species of the *Dendropanax* genus have been studied (Tables S1 and S2). 

## 4. Medicinal Uses and Pharmacological Properties of *D. morbifera*

Recently, pharmacologists, research scientists, and biologists have given considerable attention to *D. morbifera* because of reports of it having a wide-spectrum of pharmacological importance and beneficial for human health [21,22,24,53]. The leaves, roots, seeds, and stems of *D. moribifera* have been extensively used in traditional folk medicine for several diseases. Notably, the two-dimensional cell migration wound-healing assay showed significant reduction of RAoSMCs migration induced by serum in nanoparticle-synthesized leaf and stem extracts of *D. moribifera* of more than ∼50% [54]. The leaves of *D. moribifera* can be administered to reduce irritating odors and improve mouth freshness over a long period, and subsequently, microorganism growth is prevented, and storage stability is improved [35]. *D. morbifera* leaf extracts also remarkably decrease melanin content, showing potential for application as a skin-whitening agent [55], while the extracts of *D. morbifera* stems and roots have antioxidant and antiaging effects [56]. There are also reports of *D. morbifera* leaf extracts, potentially inhibiting cell proliferation in both the MDA-MB-231 and MCF-7 cell lines [57]. Song et al. reported that *D. morbifera* hot-water extracts showed significant anti-stress effects on sleep and stress-related hormones caused by chronic stress [58]. In other research, the crude extracts of *D. moribifera* leaves seemed to exhibit higher immune activation activities and were used as an immunomodulatory agent [59]. *D. morbifera* leaf extracts also appear to be a useful natural resource for the management of sexual function [60]. Meanwhile, *D. moribifera* is also used in multi-therapeutic applications. More specifically, isolated ββ-sitosterol from *D. moribifera* yielded results in terms of both skin whitening and moisturizing, and in preventing hair loss and improving hair density [61]. Hence, there is a need to evaluate the medicinal applications of *D. morbifera* through the phytochemical and pharmacological validation of both the crude extracts and compounds associated with the species. The different plant parts of *D. morbifera* have been shown by researchers to date to have a series of pharmacological activities, that is antioxidant, anti-inflammatory, anti-amnesic, neuroprotective, anti-cancer, anti-diabetic, hepatoprotective, immunomodulatory, antimicrobial, antiplasmodial, and cytotoxic functions (Table 3).

### 4.1. Antioxidant Properties of D. morbifera

The dual biological effects of reactive oxygen species (ROS) needed help to maintain normal cellular function, but also damage biological macromolecules. Excessive production of ROS results in apoptotic cell death, which can lead to the onset of various diseases and accelerate human aging [69]. It is beneficial that various chemical components sourced from plants can be used as potential antioxidants, and are considered to be promising therapeutic agents for the prevention of free-radical formation in the human body and for the treatment of various diseases, including cancer, cardiovascular disorders, neurodegenerative disorders, and other conditions [70].

#### 4.1.1. In Vitro Studies

Previous preclinical studies have shown that *D. moribifera* displays potential antioxidant activities and enhances cellular antioxidant defense systems by acting as a direct free-radical scavenger, thus reducing lipid peroxidation and various oxidative insults. Moreover, *D. morbifera* extracts displayed 2,2-dipheny-l-picrylhydrazyl (DPPH)-radical scavenging activity patterns, similar to those of butylated hydroxytoluene and vitamin C, within a range of 31.3–62.5 µg/mL. However, *D. morbifera* extracts exhibited the potentially higher DPPH-radical scavenging activity of 82.92 ± 0.49% compared to butylated hydroxytoluene, at 56.71 ± 6.34%, and vitamin C, at 90.11 ± 0.13%, respectively, in the positive control groups, at the highest concentration 500 µg/mL. These results support the existence of DPPH-radical scavenging activity of *D. morbifera* 82.92 ± 0.49% to a similar degree as that of vitamin C 90.11 ± 0.13%, at the same concentration of 500 µg/mL [35]. Youn et al. evaluated the antioxidant activities of different solvent extraction conditions such as hot water, 30% ethanol, and 60% ethanol extracts of *D. morbifera*, using DPPH-free radical scavenging, and 2, 2’-azinobis-3-ethylbenzothiazoline-6-sulphonate (ABTS) assay. The half-maximal inhibitory concentration (IC_50_) values for the DPPH assay for hot water, 30% ethanol and 60% ethanol were 6.89 mg/mL, 5.70 mg/mL, and 5.59 mg/mL, while those for ABTS for hot water, 30% ethanol and 60% ethanol were 3.79 mg/mL, 3.75 mg/mL, and 3.58 mg/mL, respectively [25]. Similar studies have indicated that antioxidant activities of different parts such as roots, leaves and stems of 95% ethanol extracts of *D. morbifera* in a 10 to 200 µg/mL treatment inhibited intracellular active oxygen production against hepatocellular injury induced by *t*- BHP. These results suggest that active component acting as an antioxidant is present in the extract; it can be shown that various parts of *D. morbifera* extracts significantly and concentration-dependently suppressed ROS production in HepG2 cells [32]. Shin et al. demonstrated the antioxidant activities of supercritical fluid stem extracts of *D. morbifera*, using DPPH and superoxide dismutase (SOD) assay in HS68 skin fibroblast cells. These results suggest that stem parts of *D. morbifera*, potentially obtained concentration-dependently, enhanced the antioxidant activity through increased the DPPH-radical scavenging activity and SOD activity [56]. 

#### 4.1.2. In Vivo Studies

In a thioacetamide-treated rat model, 50 mg/kg daily of *D. morbifera* administration for six weeks significantly decreased the hepatic malondialdehyde (MDA) content and significantly increased the glutathione (GSH) content, as well as ameliorated the catalase (CAT) and SOD activity in thioacetamide-treated rats. As such, the resultant research data suggested that *D. morbifera* intensely prevented hepatic fibrosis by inhibiting thioacetamide-induced oxidative damage [52]. Defined as the over-production of free-radical generation, diabetes plays a vital role in the growth of diabetic nephropathy. In this context, the antioxidant activity of *D. morbifera* extracts on streptozotocin-induced oxidative stress was assessed through measuring antioxidant enzyme activity in the kidneys of diabetic rats. The administration of *D. morbifera* extracts of 25 mg/kg for four weeks significantly reduction in the MDA level and prompted significant enhancements in GSH, CAT, and SOD activities in streptozotocin-induced diabetic rats [30]. Elsewhere, Kim et al. assessed the anti-amnesic effect of ethyl acetate extracts of *D. morbifera* on high-fat diet-induced diabetic mice by evaluating the level of MDA, and the oxidized GSH, SOD, and GSH contents, respectively. Treatment with ethyl acetate extracts of *D. morbifera* at 20 and 50 mg/kg significantly decreased the MDA level, and enhanced the level of SOD activity and GSH content in both liver and brain tissues. The study proven that *D. morbifera* worked as a cellular antioxidant potential through inhibiting the formation of lipid peroxidation in high-fat diet-induced diabetic mice [14]. In addition, the stem extracts of *D. morbifera* in a 100-mg/kg oral administration inhibited cadmium-induced hippocampus oxidative damage in Wistar rats, by regulating lipid peroxidation and the activities of CAT, glutathione peroxidase (GPx), Zn-superoxide dismutase (SOD1), and glutathione-S-transferase (GST) [15]. Similar studies have indicated that oral treatment with *D. morbifera* 100 mg/kg daily for 12 weeks inhibited the hypothyroidism-induced lessening of antioxidant enzymes, including SOD1, CAT, and GPx, as well as reduced the oxidative stress, as evaluated by lipid peroxidation levels in the hippocampus of the rat model [28]. In a mercury-injected rat model, the oral administration of leaf extract of 100 mg/kg of *D. morbifera* for four weeks significantly attenuated hippocampus oxidative stress by regulating the activities of SOD1, CAT, GPx, and GST, as compared with in the untreated mercury-injected group [24]. These results recommend that various parts of *D. morbifera* potentially ameliorate neurotoxin-induced oxidative damage in the hippocampus via the induction of antioxidant enzymes. In a clinical trial of 60 healthy subjects, Seo et al. reported that subjects who intake the tablets containing 300 mg *D. morbifera* leaf extracts daily for 60 days had significantly decreased serum levels of mercury, MDA, and cadmium and significant increase in SOD1 activity. The findings of their study demonstrated that chronic consumption of *D. morbifera* leaf extract can help to protects the antioxidant defense system in humans and excretion of mercury and cadmium from serum [71]. 

### 4.2. Anti-Inflammatory Properties of D. morbifera

#### 4.2.1. In Vitro Studies

Several findings have previously confirmed that extracts of *D. morbifera* have potential anti-inflammatory activities. The main mechanism involved here is thought to be the down-regulation of the protein nuclear factor kappa B (NF-κB) inflammatory signaling pathway [72]. Choo et al. demonstrated that anti-inflammatory effects of ethanol extracts from the leaves of *D. morbifera* on lipopolysaccharide (LPS)-stimulated RAW264.7 macrophages. Their study results displayed that 200 and 400 µg/mL of *D. morbifera* significantly inhibited the nitric oxide (NO) production and the lowered expression of interleukin-6 (IL-6) and tumor necrosis factor-alpha (TNF-α) in LPS-stimulated cells. Further, it was also determined that *D.* *morbifera* treatment significantly decreased NF-κB activation in the LPS-stimulated group. However, the activation of IκB-α was not significantly changed [17]. Other in vitro studies have suggested that *D. morbifera* extracts have anti-inflammatory activities. The in vitro experiments result revealed that varying methanolic extracts of *D. morbifera* (10, 25, 50, and 100-µg/mL chloroform fractions) significantly and, in a concentration-dependent manner, inhibited the production of NO, interleukin-1β (IL-1β), prostaglandin E_2_ (PGE_2_), IL-6, and TNF-α in LPS-induced RAW264.7 macrophages, with IC_50_ values of 8.2 and 14.8 µg/mL, respectively. The levels of messenger (mRNA) expression in each cytokine were also inhibited by *D. morbifera* chloroform fraction, supporting that *D. morbifera* has potential anti-inflammatory effects [26]. Hyun et al. reported that methanolic extracts collected from two different period of *D. morbifera* leaves (green and senescent leaves)*,* and its bioactive derivative phenolic compounds, such as quercetin, rutin, ferulic acid, (+)-catechin, chlorogenic acid, myricetin, and resveratrol exhibited a strong anti-inflammatory effect against LPS-induced RAW 264.7 macrophages [73]. Kim et al. reported that 1-tetradecanol, which was isolated from the n-hexane fraction of *D. morbifera,* effectively inhibits Helicobacter pylori stain-induced pro-inflammatory mediators, such as vascular endothelial growth factor (VEGF) and IL-8 in gastric epithelial cells. These results confirmed that treatment with different concentrations of 1-tetradecanol (30, 100, and 300 µM) treatments may have a potential preventive effect on gastric inflammation induced by H. pylori [74]. Meanwhile, other research suggests that oleifolioside-A, a new triterpenoid compound isolated from *D. morbifera*, suppresses LPS-induced proinflammatory cytokines and their mediators in a dose-dependent manner. More specifically, different concentrations of oleifolioside-A (0, 5, 10, and 15 μM) effectively suppressed NO, PGE_2_, inducible NO synthase (iNOS), and cyclooxygenase-2 (COX-2) in both the mRNA and protein expression, and following treatment, significantly and dose-dependently inhibited IκB-α degradation and phosphorylation and subsequent translocation of the NF-κB p65 subunit to the nucleus [75].

#### 4.2.2. In Vivo Studies

Several findings have strongly confirmed the anti-inflammatory effects of *D. morbifera* demonstrated by animal experiments. Kim et al. demonstrated that water extracts from the aerial parts of *D. morbifera* 25-mg/kg oral administration favorable anti-inflammatory effects in cisplatin-induced male Sprague–Dawley rats. It could significantly reduce the inflammatory cytokines, such as IL-1β, IL-6, and TNF-α expression. The same study also found that IL-10 expression was significantly diminished by *D. morbifera* administration in cisplatin-induced rats [65]. Separately, Sachan et al. reported that stem and leaf water extracts of *D. morbifera* on streptozotocin-induced diabetic rats and suggested that water extracts of *D. morbifera* 25 mg/kg administration suppressed proinflammatory cytokine secretion and significantly enhanced anti-inflammatory cytokine expression in diabetic rats. The authors also confirmed that extracts of *D. morbifera* markedly inhibited transforming growth factor-beta 1 (TGF-β1) expression in diabetic rats [30]. Birhanu et al. reported that oral administration of fermented leaf extracts of *D. morbifera* at 125, 250, and 500 mg/kg daily for 14 days significantly and dose-dependently reduced levels of TNF-α, various interleukins (IL-2, IL-4, IL-5, IL-6, IL-10, IL-12, IL-12p70, and IL-13), and elevated interferon-gamma (IFN-γ) in immunized BALB/C mice [27]. Taken together, both these cellular and animal experiments confirm that *D. morbifera* and its bioactive compounds have potential as novel anti-inflammatory therapeutic agents capable of targeting NF-κB signaling.

### 4.3. Action of D. morbifera on the Central Nervous System

#### 4.3.1. In Vitro

**Figure 3:**
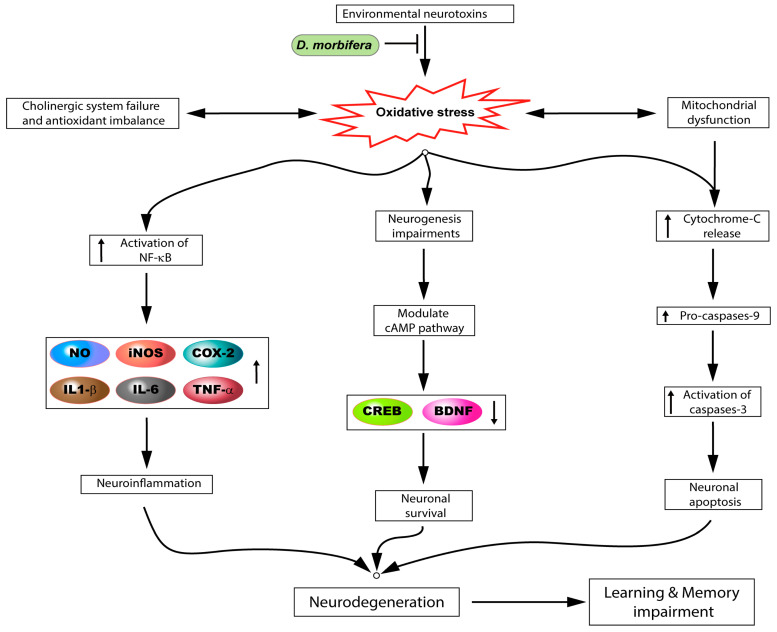
Neuroprotective and memory enhancing effect on *D. morbifera*. MAPK: mitogen-activated protein kinase; NFκB: Nuclear factor kappa B; NO: Nitric oxide; iNOS: Inducible nitric oxide synthase; COX-2: Cyclooxygenase-2; IL-1β: Interleukin 1 beta; IL-6: Interleukin 6; TNF-α: Tumor necrosis factor-alpha; cAMP: Cyclic adenosine monophosphate; CREB: cAMP response element-binding; BDNF: Brain-derived neurotrophic factor.

Several studies have previously reported the neuroprotective effect of *D. morbifera,* and its bioactive compound mechanisms of action have been proposed (Figure 3). Kim et al. evaluated the neuroprotective effects of ethyl acetate extracts of *D. morbifera* against high glucose-induced neurotoxicity in PC12 and MC-ⅨC cells. Ultimately, *D. morbifera* treatment effectively prevented oxidative damage through inhibition of acetylcholinesterase (AChE) as an acetylcholine (ACh)-hydrolyzing enzyme activity in high glucose-induced PC12 and MC-ⅨC cells [76]. A similar study also determined that the ethyl acetate extracts of *D. morbifera* at different concentrations of 10, 50, and 100 μg/mL significantly and dose-dependently inhibited mitochondrial ROS generation, mitochondrial dysfunction, and the elevation of Ca^2+^ levels, and significantly reversed, subsequently, the nuclear translocation of apoptosis-inducible factor (AIF) in glutamate-induced HT22 mouse hippocampal neuronal cells [67]. Our laboratory previously demonstrated that *D. morbifera* and its isolated compound chlorogenic acid effectively attenuated inflammatory marker expression in LPS-induced BV-2 cells. Pre-treatment with *D. morbifera* at different concentrations of 100, 250, and 500 μg/mL and administration of chlorogenic acid at 0.5, 1, and 2 mM attenuated NO production, and proinflammatory mediators, and subsequently, *D. morbifera* suppressed the phosphorylation of both the IκB-α and NF-κB p65 subunits and mitogen-activated protein kinase (MAPK) signaling in LPS-stimulated BV-2 cells [13]. In a similar study, Shim et al. demonstrated that treatment with 10 μg/mL of significantly suppressed the level of pro-inflammatory cytokines and attenuated the activation of MAPKs and NF-κB signaling. In addition, the treatment up-regulated the M2 polarization of alternative anti-inflammatory markers, while reducing the expression of the classical, pro-inflammatory M1 activation in LPS-stimulated BV-2 cells [20]. We also recently reported on the neuroprotective action of rutin, which is isolated from *D. morbifera* in SH-SY5Y cellular models of neurotoxicity induced by rotenone. Rutin administration at 1, 5, and 10 µM significantly attenuated high levels of intracellular Ca^2+^ and reduced the levels of mitochondrial membrane potential (MMP), subsequently lessening the rotenone-induced generation of ROS levels in SH-SY5Y cells. In addition, rutin treatment modulated the Bax/Bcl-2 ratio, and caspase-3 activation and prevented apoptotic cell death. Finally, it was also observed that rutin effectively suppressed JNK and p38 MAPK signaling induced by rotenone [66].

#### 4.3.2. In Vivo

Kim et al. reported the neuroprotective effect of *D. morbifera* in the context of cadmium-induced hippocampus neurotoxicity in a rat model. Their study suggests that 100 mg/kg daily of *D. morbifera* for 28 days by oral administration attenuates cadmium-induced cognitive dysfunction via an increase in AChE activity, cell proliferation and neuroblast differentiation in the hippocampus [77]. Other findings contend that leaf extracts of *D. morbifera* given at 100 mg/kg daily for four weeks significantly reduced mercury levels in hippocampus homogenates, lessened ROS generation, and reversed hippocampal activities in a dimethylmercury-induced rat model [24]. Similar findings also demonstrated that treatment with *D. morbifera* at 100 mg/kg effectively restored the hypothyroidism-induced changes in the levels of thyroxine (T4), serum triiodothyronine (T3), and thyroid-stimulating hormones in the hippocampus of rats [28]. Lee et al. evaluated the effect of *D. morbifera* 100 mg/kg daily given orally for 10 weeks to D-galactose-treated mice and observed a significantly greater enhanced in swimming speed, escape latency, and spatial memory preference. In addition, this treatment significantly attenuated the microglial activation and decrease in inflammatory cytokine expression in the rat hippocampus. The same study also observed that *D. morbifera* administration significantly increased IL-4 expression, but did not result in significant changes in the IL-10 levels in the rat hippocampus [68]. Our in vivo experiments have previously demonstrated a neuroprotective effect presented by *D. morbifera* in a mouse model of neurodegeneration induced by MPTP. Specifically, the aqueous extracts of *D. morbifera* 200 mg/kg given orally triggered improved behavioral function, inhibited neuroinflammation mediated by microglial activation, significantly attenuated neuronal loss, and subsequently restored tyrosine hydroxylase (TH) levels in MPTP-induced PD mice [13]. Kim et al. found that pretreatment with *D. morbifera* 100 µg/mL reduced the levels of intracellular ROS and cognitive damage, and protected against neuronal cell death induced by A*β*1–42 peptide. In addition, *D. morbifera* given at 100 and 300 mg/kg enhanced the cholinergic system and antioxidant levels and altered the expression of both phosphorylated cAMP response element binding protein (CREB) and brain-derived neurotrophic factor (BDNF) in the hippocampus, in an A*β*1–42 peptide-induced Alzheimer’s model [69]. In the hippocampus, diabetic mice treated with 20 and 50 mg/kg ethanol extracts of *D. morbifera* showed significantly improved glucose tolerance status, reduced behavioral impairments (e.g., Morris water maze, Y-maze and passive avoidance test), and appeared noticeably recovered, according to observations of modulating cholinergic systems, such as ACh level and the inhibition of AChE, and antioxidant systems. Additionally, significant protection against mitochondria damage was also achieved by inhibiting p-Akt, p-IRS, p-JNK, and p-tau protein expression levels [14]. Kim et al. evaluated the anti-amnesic effect of *D. morbifera* extracts 300 mg/kg daily administration for 11 weeks improved the behavioral function and significantly diminished serum insulin, glucose, and blood urea nitrogen (BUN) levels that had been increased as a result of streptozotocin-induction in a rat model. In addition, *D. morbifera* treatment potentially inhibited AChE activity and increased the ACh level in the hippocampus in streptozotocin-induced diabetic rats [78]. In cesium chloride-induced Sprague–Dawley rats, *D. morbifera* was orally administered at 30, 100, and 300 mg/kg daily for four weeks, and the results suggested that *D. morbifera* administration significantly and concentration-dependently reduced the inflammatory response in the kidneys, and increased the antioxidant enzyme levels in the hippocampus, although it did not decrease the accumulation of cesium in the blood, kidney, or liver [79]. 

### 4.4. Anti-cancer Activity of D. morbifera

**Figure 4:**
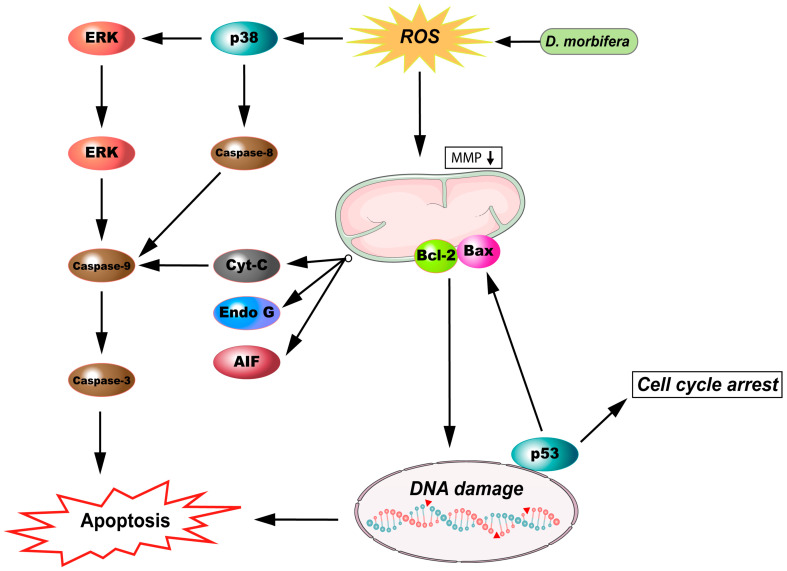
Apoptosis induced by *D. morbifera* in cancer. ROS: reactive oxygen species; MMP: Mitochondrial membrane potential; p38: P38 mitogen-activated protein kinases; ERK: Extracellular Receptor Kinase; Endo G: Endonuclease G; Cyt-C: Cytochrome-C; AIF: Apoptosis inducing factor; DNA: Deoxyribonucleic acid; Bcl2: B-cell lymphoma 2; JAK: janus kinase; p53: tumor protein.

Most chemically synthesized anti-cancer agents are extremely toxic, not only to cancer cells, it is also highly toxic to normal living cells as well. Considerable recent attention has been given to using plants as natural sources of medicinal components, especially to their bioactive compounds which may exhibit their therapeutic effect by destroying cancer cells with minimal deleterious effects to the normal cells. At present, more than 60% of currently used anti-cancer drugs have been formed from natural sources, such as medicinal plants, marine organisms, and microorganisms [80]. Meanwhile, vinca alkaloids, vincristine, and vinblastine have also been used to treat cancer and the developed as possible anti-cancer agents based on previous scientific studies that revealed that different classes of medicinal plant products, including flavonoids, polyacetylene, and alkaloids show potential as anti-cancer agents [81,82]. Recently, numerous polyacetylene compounds isolated from *D. morbifera* leaves with potential anticomplement and anti-cancer effects have showed cytotoxic activity against different tumor cell lines (L-1210, H-4IIE, Hep-G2, and A-431), but not against normal hepatocytes [11,53]. In this section, a comprehensive overview of the effects of the *D. morbifera* species and its active components in various cancer experiments is presented and discussed (Figure 4).

#### In Vitro and In Vivo Studies

The methanol extracts of yellow leaves, green leaves, and debarked stems of *D. morbifera* were evaluated by analyzing potential anti-cancer activity for different tumor cell lines, including COLO-205, HOS, SNU-245, SNU-308, Huh-BAT, and Huh-7. In particular, the authors investigated the possible mechanism of action behind the impact of *D. morbifera* extracts on the most vulnerable cell line Huh-7. Based on the investigation, *D. morbifera* extracts at 50 µg/mL caused an increase in apoptotic and triggering p53 and p16 in Huh-7 cells. Moreover, the extracts of the green leaf and the debarked stems of *D. morbifera* significantly inhibited the activation of ERK and Akt levels, resulting in the suppression of Huh-7 cell proliferation [21]. Aceituno et al. demonstrated that novel synthesis of silver nanoparticles (Ag-NPs) using leaves extract of *D. morbifera* showed cytotoxicity in the cell lines A549 and HepG2. Cells treated with synthesized Ag-NPs at 25 µg/mL displayed significant increases in ROS production, reduction in cell migration, and cell apoptosis in both cell lines. Moreover, synthesized Ag-NPs treatment resulted in significant modulation of the EGFR/p38 expressions in A549 cells [70]. Elsewhere, Lee et al. evaluated the proapoptotic activities of ethanol extracts of *D. morbifera* stem bark on human leukemia U937 cells. The extracts of *D. morbifera* at various concentrations of 0, 20, 40, 60, 80, and 100 µg/mL exhibited decreased cell growth, due to inhibited cell proliferation, the induction of cell apoptosis, and the downregulation of anti-apoptotic IAP family proteins, and together with the subsequent inhibition of Bcl-2 and Bcl-xL protein expression levels, the loss of MMP, and bid cleavage, suggesting that the apoptosis of U937 cells occurred through both intrinsic and extrinsic pathways [71]. In the study by Jin et al., oleifolioside-B, a triterpene glycoside compound isolated from the stem of *D. morbifera*, was evaluated in A549 cells. Here, oleifolioside-B exposure at 30 µM induced caspase activation and typical apoptotic features. Subsequently, oleifolioside-B treatment increased autophagy, as indicated by an increase in the levels of the autophagy-specific gene MAP1LC3 and Atg3, which was associated with the inhibition of Nrf2 expression [83]. Similar studies have also reported on oleifolioside-A, a new anticancer agent isolated from *D. morbifera,* and found that the mechanisms of oleifolioside-A exhibited activities of potentially induced caspases activation and apoptotic features in HeLa cells. In addition, oleifolioside-A administration induced the up-regulation of the loss of MMP, Bad, AIF, and EndoG factors, and apoptosis [84]. Meanwhile, Lee et al. evaluated the anticancer activity of dendropanoxide isolated from the leaves and stems of *D. morbifera* and the results study indicated treatment with 60 µM could modulate the autophagy through ERK1/2 activation in human osteosarcoma (OS) cells and induce apoptosis [29]. Recently, using an in vivo tumor xenograft model, the administration of *D. morbifera* 25 mg/kg orally daily for 10 days achieved renoprotective properties against cisplatin-induced nephrotoxicity without blocking the chemotherapeutic efficacy. The authors suggested that *D. morbifera* might be combined with cisplatin therapy to protect renal function during the treatment of solid tumor patients [65]. 

### 4.5. Anti-Diabetic Activity of D. morbifera

Diabetes is a metabolic disorder that causes high blood sugar and insufficient secretion of insulin in the body, resulting in hyperglycemia, which may consequently lead to stroke, amputation, kidney failure, heart attack, or heart failure [85]. The worldwide number of people suffering from diabetes is expected to rise from 285 million in 2000 to 439 million in 2030 [86]. Hyperlipidemia and hyperglycemia are mainly involved in the development of diabetic complications, which are the major factors behind diabetic-associated morbidity and mortality [87]. Recently, a number of scientific reports have suggested the beneficial impacts of plant-derived natural products on the attenuation of diabetic complications through an anti-diabetic mechanism of action with fewer side effects [88]. 

#### In Vitro and In Vivo Studies

Obesity is a complex disease. It is linked with several metabolic complications, including insulin resistance, hypertension, glucose intolerance, cardiovascular disease, type 2 diabetes, and dyslipidemia. Herein, the authors reported that water extracts of *D. morbiferus* demonstrated no toxicity at a concentration ranging from 5 to 500 μg/Ml in 3T3-L1 cells. It has also been suggested that *D. morbiferus* might ensure improved cell viability and may trigger reduction in intracellular triglyceride level and glucose uptake. Furthermore, *D. morbiferus* treatment inhibits adipogenesis-related genes in both protein and mRNA expression, suggesting the presence of anti-obesity effects, especially also given an earlier report confirmed that *D. morbiferus* is a cholesterol-lowering agent [9].

**Figure 5:**
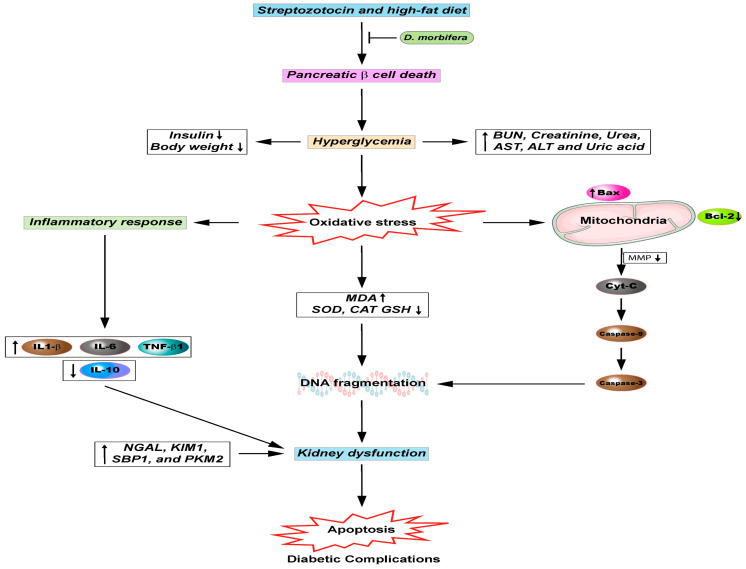
Anti-diabetic effect on *D. morbifera*. Bcl2: B-cell lymphoma 2; MMP: Mitochondrial membrane potential; Cyt-C: Cytochrome-C; IL-1β: Interleukin 1 beta; IL-10: Interleukin 10; IL-6: Interleukin 6; TGF-β1: Transforming growth factor beta 1; NGAL: KIM1: Kidney injury molecule-1; Neutrophil gelatinase-associated lipocalin; SBP1: Selenium binding protein-1; PKM2: Pyruvate kinase muscle isozyme M2; BUN: Blood urea nitrogen; ALT: Alanine amino transferase; AST: Aspartate amino transferase; MDA: Malondialdehyde; DNA: Deoxyribonucleic acid; CAT: Catalase; SOD: Superoxide dismutase; GSH: Reduced glutathione.

Sachan et al. recently confirmed the protective effects of leaf and stem extracts of *D. morbifera* against streptozotocin-induced renal fibrosis. However, *D. morbifera* water extracts at 25 mg/kg administration protected body and organ weight loss and significantly increased serum BUN and creatinine levels, antioxidant enzyme activity, and oxidative stress parameters in diabetic rats. In addition, *D. morbifera* extracts protected against histopathological damage in the kidney and pancreas in a diabetic rat model, and caused a subsequent significant reduction in microalbumin, KIM-1, SBP1, and PKM2 levels in the urinary excretion of diabetic rats following the administration of *D. morbifera*. Additionally, the water extracts of *D. morbifera* significantly reduced proinflammatory cytokines and fibrosis markers in the kidney of streptozotocin-induced diabetic rats [30]. Meanwhile, the methanol leaf extracts of *D. morbifera* at 30, 60, and 100 mg/kg daily given orally for 14 days achieved significant and concentration-dependent hypoglycemic activity, by significantly decreasing the total cholesterol, serum glucose, urea, triglyceride, creatinine, uric acid, and alanine amino transferase (ALT), and aspartate amino transferase (AST) levels in streptozotocin-induced diabetic rats. Interestingly, the anti-diabetic activities of *D. morbifera* was more effective than that observed with the use of glibenclamide 600 µg/kg, a known anti-diabetic agent [22]. Earlier studies have reported the anti-diabetic activities of ethyl acetate fraction of *D. morbifera* administered at 20 and 50 mg/kg, which significantly enhanced glucose tolerance, and regulated antioxidant enzyme levels in high-fat diet-induced diabetic mice. The study suggested that *D. morbifera* treatment protected against abnormal activity of mitochondria, improved p-JNK, p-IRS, p-Akt, and inhibited p-tau protein expression. Finally, luteolin-7-O-rutinoside, rutin, isoorientin, and orientin were identified as the main phenolic compounds of ethyl acetate fraction of *D. morbifera* using ultra-performance liquid chromatography/quadrupole time of flight tandem mass spectrometry (UPLC-QTOF/MS) analysis [14]. Other studies reported that the administration of *D. morbifera* at 50, 100, and 200 mg/kg concentration-dependently stimulated AMP-activated protein kinase in the liver, adipose tissue, and skeletal muscle, leading to the development of protein and gene expressions related to insulin resistance and glucose homeostasis in db/db mice [89]. Interestingly, Young et al. showed that the four extracts, including the water extracts of the leaves and stem and ethanol extracts of the leaves and stem, of *D. morbifera* administered at 25 to 100 µg/kg maintained body weight, enhanced insulin concentration, and reduced the glucose concentration in the blood in streptozotocin-induced diabetic mice [31]. The overall study results support that *D. morbifera* and its bioactive ingredients show potential as anti-diabetic active agents for inclusion in anti-hyperglycemia and anti-hyperlipidemia therapies (Figure 5). 

### 4.6. Hepatoprotective and Immunomodulatory Effects of D. morbifera

Liver disorders are a leading cause of concern worldwide and available medical treatment options are potentially insufficient. As such, a huge number of plant extracts and their bioactive compounds have been proposed to have significant hepatoprotective activity or to be treatment agents [90]. The ethanolic root, leaves, and stem extracts of *D. morbifera* at concentrations of 50, 100, 150, and 200 μg/mL exhibited strong antioxidant properties, showed hepatoprotective activity against t-butyl hydroperoxide-induced HepG2 cells, reduced ROS generation, and lowered the mRNA level of COX-2 in LPS-stimulated Raw246.7 cells [32]. Elsewhere, *D. morbifera* and its isolated compounds, chlorogenic acid and rutin, were investigated against alcohol-induced hepatotoxicity in the human liver cancer HepG2 cell line model. *D. morbifera* at different concentrations 12.5, 25, and 50 μg/mL was reported to protect from liver injury by scavenging the ROS generation in chronic alcoholism, and was proven to be useful as a functional food product supplement to stop liver injury caused by excessive alcohol consumption [91]. The in vivo experimental results showed that the aqueous extracts of *D. morbifera* administered at 100 and 300 mg/kg resulted in the prevention of ethanol-induced hepatotoxicity due to reductions in serum AST and ALT levels, and by maintaining enzymatic oxidant status and suppressing cytochrome P-450 2E1 expression. In addition, *D. morbifera* enhanced alcohol dehydrogenase activities and reduced blood ethanol concentrations in alcohol-induced hepatocyte-injured rats [33]. The results suggested that *D. morbifera* and its primary active constituents were a source of antioxidant and hepatoprotective properties, and indicated their potential as therapeutic agents.

Recently, several findings have highlighted that extracts from plants play a vital role in the disease prevention and cure of several infectious diseases by modulating and maintaining the immune system. Therefore, their use and applications has growing dramatically [92,93]. In addition, plant-sourced traditional medicines regulate the immune system through either stimulating or suppressing adaptive or innate immune cells/molecules [94]. Immune system function is closely related to normal human health and plant-based immunomodulatory bioactive compounds treat several infection diseases, by enhancing the natural immune resistance of our body [95]. Hence, several studies to date have recorded the immunomodulatory effect of *D. morbifera* extracts in both cellular and animal models.

Recently, Birhanu et al. reported that *D. morbifera* (125, 250, and 500 mg/kg) administration orally daily for 14 days triggered an increase in spleen cells in mice. In addition, T-cell phenotypic investigation indicated that *D. morbifera*-treated groups showed higher CD8a+, CD11b, and CD3+ T-cell expression, and the significant suppression of inflammatory cytokines. Moreover, the IgG super-family was significant downregulated in a concentration dependent manner by *D. morbifera* administration of 4.5%, up to 43.7% [27]. The growing level of splenic B- and T-cells upon treatment with *D. morbifera* plant extracts suggests a T-cell-mediated immunomodulatory and anti-inflammatory effect on the immune system.

Numerous signaling pathways are mainly involved in T-cell activation and the expression and proliferation of inflammatory cytokines, including IL-2 and IFN-γ. IL-2 plays an important role in early T lymphocyte clonal diversity and development. Important transcription factors, such as AP-1, NF-AT, and NF-κB, are mainly involved in IL-2 production in T-cells [96]. Oxidant-mediated tissue injury is a specific hazard to the immune response, and is closely related to ROS generation, leading to a reduction in the antioxidant defense system. Meanwhile, oxidative stress-mediated tissue injury is associated with immune shortages, resulting in decreased levels of total lymphocytes, IL-2 production, and T-cell subsets [97]. In contrast, the ethyl acetate fraction of *D. morbifera* water extracts significantly enhanced the production of IL-2 and IFN-γ cytokines in activated T-cells and splenocytes, and elevated the NF-AT transcriptional activity of EL-4 T-cells, but no changes were observed in the NF-κB promoter. Therefore, the strong antioxidant effects of *D. morbifera* could synergistically contribute to its immune enhancing and/or modulating effect, by regulating the IL-2 concentration, total lymphocyte count, and T-cell activation [98].

The immunomodulatory effect of *D. morbifera* branch water extracts increased the production of pro-inflammatory cytokines and the inflammatory mediators in RAW264.7 cells. Moreover, *D. morbifera* was involved in the activation of NF-κB signaling, as well as the phospho-IκBa. The translocation of p65, a subunit of NF-κB, was also increased in RAW264.7 cells. In addition, an in vivo study demonstrated that *D. morbifera* given orally at 500, 1000, and 2000 mg/kg increased splenocyte cytokines, NO production, and lactate dehydrogenase (LDH) significantly in a concentration-dependent manner, as compared to the control group. Collectively, the results of this study suggested that *D. morbifera* extracts enhanced innate immune response by modulation of NF-κB signaling, leading to the up-regulation of pro-inflammatory cytokines, and acted as a therapeutic in the context of immune stimulation [34].

### 4.7. Antimicrobial, Antiplasmodial, and Anticomplementary Activities of D. morbifera

Several studies have previously revealed the antibacterial activity of *D. morbifera* extracts on gram-negative and gram-positive bacterial strains. Kim et al. previously evaluated the antimicrobial activities of *D. morbifera* extracts, using chloroform, ethyl acetate, n-hexane, and butanol solvent using a paper disc test against *Candida albicans* and *Streptococcus mutans*. Both the n-hexane and butanol solvent fractions among the four types showed potential antimicrobial activity at *D. morbifera* extract concentrations of 40, 80, and 100 μg/mL against *S. mutans* and *C. albicans*, while observations made at a 20-μg/mL concentration did not show a clear antibiotic effect against *S. mutans* [35].

Chung et al. assessed the antiplasmodial activities of oleifoliosides A and oleifoliosides B methanol extracts from *D. morbifera* stem bark, using an in vitro, semi-automated micro-dilution assay that was observed using a chloroquine-sensitive strain of *Plasmodium falciparum* (D10). Artemisinin and chloroquine were used as the normal standard drugs for the antiplasmodial activity assay. Results from the in vitro antiplasmodial activity assay confirmed that oleifoliosides A and oleifoliosides B showed significant antiplasmodial activity, with IC_50_ values of 6.2 and 5.3 mM, respectively [36]. 

The human complementary system plays a essential role in the host’s defense against overseas invasive organisms, including bacteria, fungi, and viruses, as well as an external infection of the wound. However, activation of this system may lead to inflammatory and degenerative pathological reactions, such as systemic lupus, dermatological disorders, Sjogren’s syndrome, rheumatoid arthritis, erythematosus, multiple sclerosis, and gout [99]. Chung et al. evaluated the anticomplementary activity of (3S)-diynene, (3S,8S)-falcarindiol and (3S)-falcarinol aqueous leaves extracts using the CCl4 fraction of *D. morbifera*. The hemolytic assay results demonstrated that (3S)-diynene, (3S, 8S)-falcarindiol, and (3S)-falcarinol isolated from the leaves of *D. morbifera* exhibited complementary activity IC_50_ values of 39.8 mM, 15.2 mM, and 87.3 mM respectively [53]. Meanwhile, Park et al. evaluated the anticomplementary activities of (9Z, 16S)-16-Hydroxy-9,17-octadecadiene-12,14-diynoic acid isolated from methanol extracts of *D. morbifera* leaves and found that isolated (9Z, 16S)-16-Hydroxy-9,17-octadecadiene-12,14-diynoic acid from *D. morbifera* exhibited significant effects on the classical pathway of the complement system with an IC_50_ value of 56.98 µM as compared with IC_50_ value of 70.09 µM for tiliroside, a standard drug used as the control [47]. 

### 4.8. Cytotoxicity and Toxicity Effects of D. morbifera

Kim et al. evaluated the cytotoxicity of methanolic extracts of *D. morbifera* leaves against different tumor cell lines, including endometrial, colon, ovarian, and cervical cancer cell lines. Treatment with the methanolic extracts of *D. morbifera* at 50 and 100 μg/mL had cytotoxic effects on these four types of cancer cell lines showed cytotoxicity of more than 93% at doses greater than 100 μg/mL and 6 to 11% of cytotoxicity at doses of less than 50 μg/mL, as compared with in normal cell lines [100]. Wang et al. evaluated the cytotoxic activities of synthesized silver nanoparticles (AgNPs) and gold nanoparticles (AuNPs) from *D. morbifera* leaf extracts for potential anti-cancer activity in a human keratinocyte cell line and A549 human lung cancer cell line and, in this context, both synthesized nanoparticles exhibited anti-cancer activities. D-AgNPs 100 µg/mL showed potent cytotoxic effects after 48 hours on the lung cancer cells but not the human keratinocyte cell line, whereas D-AuNPs did not exhibit cytotoxic effects on either cell line at the same concentration [38]. Kim et al. evaluated the cytotoxicity of *D. morbifera* extracts by analysis with a 3-(4,5-dimethylthiazol-2-yl)-2,5-diphenyltetrazolium bromide (MTT) assay, using normal human HNOK cells. Treatment with *D. morbifera* extracts 2.5 to 10 μg/mL retained a high level of cell viability of 60% to 100%, without affecting the cell survival rate relative to in the control group. Concentrations of *D. morbifera* extracts ranging from 25 to 40 μg/mL demonstrated a cell survival rate of approximately 70% [35]. Park et al. evaluated the cytotoxicity activities of methanol extracts of *D. morbifera* leaves against HT22 mouse hippocampal neuronal cells left otherwise untreated as well as the toxicity induced by glutamate using the MTT assay. The exposure of the HT22 cells to 4 mM of glutamate for 24 hours resulted in a decrease in cell viability. The extracts exhibited low levels of cytotoxicity with IC_50_ values exceeding 50 µg/mL per the MTT assay. Concentration-dependent pre-treatment with extracts resulted in the maintenance of up to 80% of cell viability induced by glutamate neurotoxicity [67]. Recently, our study explored the cytotoxicity of extracts of *D. morbifera* leaves against BV-2 cells left otherwise untreated, as well as the toxicity induced with LPS using the MTT assay. Exposure of the BV-2 cells to 200 ng/mL of LPS alone or in combination with *D. morbifera* at 100, 250, and 500 μg/mL did not show any significant toxic effects. Interestingly, however, inclusion of a higher concentration of 500 μg/mL of *D. morbifera* similarly showed no toxic effects, but maintained up to 100% of the cell viability induced by LPS toxicity [13]. Separately, Yun et al. evaluated the in vitro and in vivo toxicity of leaf extracts of *D. morbifera* in a two-week and the other a 13-week repeated-dose toxicity study using Sprague–Dawley rats. The rats were given oral doses of *D. morbifera* 500, 1000 and 2000 mg/kg body weight. An assessment of the sub-chronic toxicity of these *D. morbifera* extracts given by oral administration in the rats showed potentially treatment-related results, with respect to mortality, hematology, body weight, organ weight, food/water consumption, serum biochemistry, urinalysis, necropsy, and histopathology, at varying doses of 500, 1000, and 2000 mg/kg body weight. Meanwhile, no adverse effects of *D. morbifera* extracts in the 13-week sub-chronic toxicity study were observed, which was considered to be the result of the use of only up to a 2000 mg/kg body weight dose in the rats [37]. 

## 5. Conclusions

*Dendropanax* plant parts have been consumed in various quantities and forms by numerous populations around the world for a long period, and no toxicity has been reported. The major edible parts of *Dendropanax* spp., are the leaves, which produce edible flour that is used in manufacturing cosmetics, and which is used to development of food products. In addition, recent pharmacological studies primarily focused on six *Dendropanax* spp., namely *D. morbifera, D. gonatopodus, D. Dentiger, D. capillaris*, *D. chevalieri,* and *D. arboreus.* Among these species, only one to date has achieved broad medicinal applications, including treating infectious microbial diseases, asthma, and inflammatory disorders. The in vitro and in vivo biological screening of *D. morbifera* revealed this species to be a promising source of a broad-spectrum of bioactivities, such as antioxidant, antimicrobial, and anti-inflammatory effects. Particularly, the extracts to date have showed less to moderate cytotoxic activity. *Dendropanax* plant species contain more than 259 bioactive compounds, including polyphenols, flavonoids, tannins, pyrimidines, essential oils, terpenoids, phenol carboxylic acids, and alkaloids were identified. Moreover, economic and commercial importance of these plant phytochemicals had proven to be suitable for cosmetics, food industries, agricultural, pharmaceutical applications. 

Numerous ethnopharmacological indications of this medicinal plant have been confirmed based on scientific investigations and their reports. Considering the extensive variety of pharmacological actions as well as significant large number of bioactive active photochemical agents, *D. morbifera* could be a good candidate to involved in future initiative in drug discovery. Considering the variety of confirmed therapeutic effects, forthcoming pharmaceutical and pharmacological investigations must focus on using isolated bioactive components to develop real drugs from this plant. Additional experimental and clinical investigations are recommended to reveal the beneficial therapeutic and safety assets of *D. morbifera* and their bioactive constituents, as complementary and alternative therapeutics for the management of several disorders.

To summarize, this study provides first time up-to-date information on the potentially active phytochemicals, plant parts, extraction method, and number of active compounds present in the raw materials of *D. morbifera* and other *Dendropanax* species. This review presented the most recent in vitro and in vivo studies on *D. morbifera* to provide greater accessibility to the established experimental and clinical data. Finally, this review elucidates the importance of *Dendropanax* spp., and provides guidance for the future development of the compounds with medicinal value. The medicinally active compounds found in *D. morbifera* parts such as roots, dried barks, leaves, and stem, as a novelty, this review focuses on the collection and preparation of plants, the extraction of active compounds, and the bioactive molecules present in the plant raw material. Moreover, the study claims its advantage by providing comprehensive references for the new drug lead research from *D. morbifera* and other species.

## Figures and Tables

**Table 1:** Phytochemical constituents identified from *D. morbifera.*

Serial. No	Compound	Molecular Formula	Plant	Plant Part	Extraction Method	Content in the Extract	References
	*D. morbifera*						
1	(9Z,16S)-16-hydroxy-9,17-octadecadiene-12,14-diynoic acid	C_18_H_24_O_3_	*D. morbifera*	Leaves	Methanol	1.1 g/1.1 kg	[47]
2	Neochlorogenic acid	C_16_H_18_O_9_	*D. morbifera*	Leaf and stems	Water	656 mg/100 g	[30]
3	Stigmasterol	C_29_H_48_O	*D. morbifera*	Aerial parts	Methanol	490.7 mg/5 kg	[48]
4	β-Amyrin	C_30_H_50_O	*D. morbifera*	Aerial parts	Methanol	150 mg/1.1 kg	[47]
5	Epifriedelanol	C_30_H_52_O	*D. morbifera*	Aerial parts	Methanol	120 mg/5 kg	[48]
6	1-Tetradecanol	C_14_H_30_O	*D. morbifera*	Stems and leaves	Methanol	98.6 mg/2.5 kg	[51]
7	Quercetin 3β-gentiobioside	C_27_H_30_O_17_	*D. morbifera*	Stems and leaves	Methanol	80 mg/2.5 kg	[51]
8	α-Glutinol	C_30_H_50_O	*D. morbifera*	Leaves	Methanol	49 mg/1.1 kg	[47]
9	1-cyclohex-20,50-dienyl 1-cyclohexylethanol-*O*-β-d-xylopyranoside	C_19_H_31_O_5_	*D. morbifera*	Dried barks	Acetone	41 mg/558 g	[50]
10	Glyceryl-1, 3-dipalmito-2-olein	C_53_H_100_O_6_	*D. morbifera*	Dried barks	Acetone	41 mg/558 g	[50]
11	Oleyl-*O*-β-d-xyloside	C_23_H_43_O_6_	*D. morbifera*	Dried barks	Acetone	38 mg/558 g	[50]
12	*Cis*-6-oxogeran-4-enyl-10-oxy-*O*-β-arabinopyranosyl-4′-*O*-β-arabinopyranosyl-2”-octadec-9”’,12”’,15”’-trienoate	C_38_H_63_O_11_	*D. morbifera*	Dried barks	Acetone	32 mg/558 g	[50]
13	Linolenyl-*O*-β-D-arabinofuranoside	C_23_H_38_O_6_	*D. morbifera*	Dried barks	Acetone	31 mg/558 g	[50]
14	n-tetradecanyl oleate	C_32_H_63_O_2_	*D. morbifera*	Dried barks	Acetone	30 mg/558 g	[50]
15	Icariside D1	C_23_H_26_O_10_	*D. morbifera*	Stems and leaves	Methanol	30 mg/2.5 kg	[51]
16	α-Amyrin	C_30_H_50_O	*D. morbifera*	Aerial parts	Methanol	30 mg/1.1 kg	[47]
17	Geranilan-8-oxy-*O*-α-d-xylopyranosyl-20-n-octadec-9”,12”,15”-trienoate	C_33_H_59_O_6_	*D. morbifera*	Dried barks	Acetone	29 mg/558 g	[50]
18	Geran-3(10)-enyl-1-oxy-*O*-β-arabinopyranosyl-4′-*O*-β-arabinopyranosyl-2”-octadec-9”’, 12”’,15”’-trienoate	C_38_H_65_O_10_	*D. morbifera*	Dried barks	Acetone	28 mg/558 g	[50]
19	Guaiacol-*O*-β-D-arabinopyaranoside	C_12_H_17_O_6_	*D. morbifera*	Dried barks	Acetone	26 mg/558 g	[50]
20	β-Sitosterol	C_29_H_50_O	*D. morbifera*	Aerial parts	Methanol	25.6 mg/1.1 kg	[47]
21	*trans*-Phytol	C_20_H_40_O	*D. morbifera*	Leaves	Methanol	22 mg/1.1 kg	[47]
22	n-octadec-9,12-dienoyl-*O*-β-D-arabinopyranoside	C_23_H_40_O_6_	*D. morbifera*	Dried barks	Acetone	21 mg/558 g	[50]
23	Citroside A	C_19_H_30_O_8_	*D. morbifera*	Stems and leaves	Methanol	20 mg/2.5 kg	[51]
24	Chlorogenic acid	C_16_H_18_O_9_	*D. morbifera*	Branch	Methanol	19.5 mg/g	[10]
25	Friedelin	C_30_H_50_O	*D. morbifera*	Aerial parts	Methanol	14.5 mg/5 kg	[48]
26	(3S,8S)-falcarindiol	C_17_H_24_O_2_	*D. morbifera*	Stems and leaves	Methanol	13 mg/2.5 kg	[51]
27	Uridine	C_9_H_12_N_2_O_6_	*D. morbifera*	Stems and leaves	Methanol	11.7 mg/2.5 kg	[51]
28	Lutexin	C_21_H_20_O_11_	*D. morbifera*	Stems and leaves	Methanol	10.3 mg/2.5 kg	[51]
29	Dendropanoxide	C_30_H_50_O	*D. morbifera*	Aerial parts	Methanol	10 mg/5 kg	[48]
30	Tetradecanol	C_14_H_30_O	*D. morbifera*	Stems and leaves	Methanol	10 mg/2.5 kg	[51]
31	Rutin	C_27_H_30_O_16_	*D. morbifera*	Dried aerial parts	Water	6.38 mg/g	[13]
32	Nikoenoside	C_16_H_24_O_9_	*D. morbifera*	Stems and leaves	Methanol	5.2 mg/2.5 kg	[51]
33	Syringaresinol β-D-glucoside	C_28_H_36_O_13_	*D. morbifera*	Stems and leaves	Methanol	4.8 mg/2.5 kg	[51]
34	Isotachioside	C_13_H_18_O_8_	*D. morbifera*	Stems and leaves	Methanol	4.0 mg/2.5 kg	[51]
35	Scorzonoside	C_27_H_34_O_12_	*D. morbifera*	Stems and leaves	Methanol	3.7 mg/2.5 kg	[51]
36	Oleifolioside-B	C_45_H_76_O_17_	*D. morbifera*	Stems and leaf	Methanol	3 mg/0.3 g	[36]
37	Olean-12-en-3,24 β-diol	C_30_H_50_O_2_	*D. morbifera*	Stems and leaves	Methanol	3 mg/2.5 kg	[51]
38	Methyl chlorogenate	C_17_H_20_O_9_	*D. morbifera*	Stems and leaves	Methanol	2.7 mg/2.5 kg	[51]
39	Oleifolioside-A	C_45_H_76_O_17_	*D. morbifera*	Stems and leaf	Methanol	2.5 mg/0.3 g	[36]
40	(1*R*,2*S*)-1-(4-hydroxy-3-methoxyphenyl)-2-[4-[(1*E*)-3-hydroxy-1-propen-1-yl]-2-methoxyphenoxy]-1,3-propanediol	C_20_H_24_O_7_	*D. morbifera*	Stems and leaves	Methanol	2.4 mg/2.5 kg	[51]
41	Hyuganoside IIIa	C_26_H_34_O_12_	*D. morbifera*	Stems and leaves	Methanol	2.0 mg/2.5 kg	[51]
42	Rel-(1*R*,2*R*)-1-(4-hydroxy-3-methoxyphenyl)-2-[4-[(1*E*) -3-hydroxy-1-propen-1-yl]-2-methoxyphenoxy]-1,3-propanediol	C_19_H_24_O_7_	*D. morbifera*	Stems and leaves	Methanol	2.0 mg/2.5 kg	[51]
43	Protocatechuic acid	C_7_H_6_O_4_	*D. morbifera*	Stems and leaves	Methanol	1.8 mg/2.5 kg	[51]
44	Gastrodin	C_13_H_18_O_7_	*D. morbifera*	Stems and leaves	Methanol	1.8 mg/2.5 kg	[51]
45	Syringylglycerol 2-O-β-d-glucopyranoside	C_28_H_36_O_13_	*D. morbifera*	Stems and leaves	Methanol	1.4 mg/2.5 kg	[51]
46	Citroside B	C_19_H_30_O_8_	*D. morbifera*	Stems and leaves	Methanol	1.2 mg/2.5 kg	[51]
47	Nicotinic acid	C_6_H_5_NO_2_	*D. morbifera*	Stems and leaves	Methanol	1.2 mg/2.5 kg	[51]
48	Adenosine	C_10_H_13_N_5_O_4_	*D. morbifera*	Stems and leaves	Methanol	1.0 mg/2.5 kg	[51]
49	Thymidine	C_10_H_14_N_2_O_5_	*D. morbifera*	Stems and leaves	Methanol	1.0 mg/2.5 kg	[51]
50	Hesperidin	C_28_H_34_O_15_	*D. morbifera*	Branch	Methanol	1576.65 µg/g	[10]
51	Syringin	C_17_H_24_O_9_	*D. morbifera*	Dried aerial parts	Water	450.5 µg/g	[52]
52	Catechin	C_15_H_14_O_6_	*D. morbifera*	Branch	Methanol	359.07 µg/g	[10]
53	2,5-Dihydroxybenzoic acid	C_7_H_6_O_4_	*D. morbifera*	Branch	Methanol	259.05 µg/g	[10]
54	Salicylic acid	C_7_H_6_O_3_	*D. morbifera*	Branch	Methanol	248.91 µg/g	[10]
55	Vitexin	C_21_H_20_O_10_	*D. morbifera*	Branch	Methanol	200 µg/g	[13]
56	Quercetin	C_15_H_10_O_7_	*D. morbifera*	Branch	Water	100 µg/g	[13]
57	Myricetin	C_15_H_10_O_8_	*D. morbifera*	Branch	Methanol	97.35 µg/g	[10]
58	4-Hydroxybenzoic acid	C_7_H_6_O_3_	*D. morbifera*	Branch	Methanol	70.57 µg/g	[10]
59	Resveratrol	C_14_H_12_O_3_	*D. morbifera*	Branch	Methanol	66.23 µg/g	[10]
60	Tricin	C_17_H_14_O_7_	*D. morbifera*	Branch	Methanol	60 µg/g	[13]
61	Naringin	C_27_H_32_O_14_	*D. morbifera*	Branch	Methanol	53.19 µg/g	[10]
66	Syringic acid	C_9_H_10_O_5_	*D. morbifera*	Branch	Methanol	42.13 µg/g	[10]
63	Trans-Ferulic acid	C_10_H_10_O_4_	*D. morbifera*	Branch	Methanol	34.87 µg/g	[10]
64	Caffeic acid	C_9_H_8_O_4_	*D. morbifera*	Branch	Methanol	31.96 µg/g	[10]
65	Luteolin	C_15_H_10_O_6_	*D. morbifera*	Branch	Methanol	20 µg/g	[13]
66	Kaempferol	C_15_H_10_O_6_	*D. morbifera*	Branch	Methanol	20 µg/g	[13]
67	Gallic acid	C_7_H_6_O_5_	*D. morbifera*	Branch	Methanol	17.37 µg/g	[10]
68	p-Coumaric acid	C_9_H_8_O_3_	*D. morbifera*	Branch	Methanol	2.11 µg/g	[10]
69	trans-Cinnamic acid	C_9_H_8_O_2_	*D. morbifera*	Branch	Methanol	0.61 µg/g	[10]
70	Luteolin-7-O-rutinoside	C_27_H_30_O_15_	*D. morbifera*	Roots	Methanol	N/A	[14]
71	Isoorientin	C_21_H_20_O_11_	*D. morbifera*	Roots	Methanol	N/A	[14]
72	Orientin	C_21_H_20_O_11_	*D. morbifera*	Roots	Methanol	N/A	[14]
73	Carnosol	C_20_H_26_O_4_	*D. morbifera*	Leaves	Ethanol	N/A	[49]
74	Dextromethorphan	C_18_ H_25_ N O	*D. morbifera*	Leaves	Ethanol	N/A	[49]
75	Cannabidiol	C_21_ H_30_ O_2_	*D. morbifera*	Leaves	Ethanol	N/A	[49]
76	(−)-Bremazocine	C_20_ H_29_ N O_2_	*D. morbifera*	Leaves	Ethanol	N/A	[49]
77	Doxapram	C_24_ H_30_ N_2_ O_2_	*D. morbifera*	Leaves	Ethanol	N/A	[49]
78	Resolvin D2	C_22_ H_32_ O_5_	*D. morbifera*	Leaves	Ethanol	N/A	[49]
79	Procyclidine	C_19_ H_29_ N O	*D. morbifera*	Leaves	Ethanol	N/A	[49]
80	2-arachidonoylglycerol	C_23_ H_38_ O_4_	*D. morbifera*	Leaves	Ethanol	N/A	[49]
81	Eplerenone	C_24_ H_30_ O_6_	*D. morbifera*	Leaves	Ethanol	N/A	[49]

**Table 2:** The main bioactive compounds of *D. morbifera* and its pharmacological activities.

Compound	Molecular Structure	Plant Species	Activity Tested
Quercetin	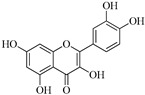	*D. morbifera*	Antioxidant, anti-inflammatory and neuroprotective
Chlorogenic acid	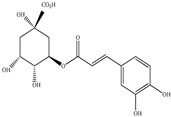	*D. morbifera*	Antioxidant and anti-inflammatory
Rutin	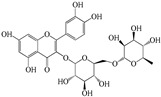	*D. morbifera*	Antioxidant, anti-inflammatory and neuroprotective
Carnosol	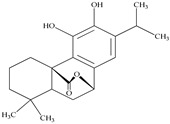	*D. morbifera*	Anti-inflammatory
Dextromethorphan	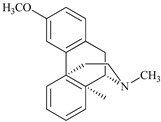	*D. morbifera*	Antioxidant, anti-inflammatory and neuroprotective
Cannabidiol	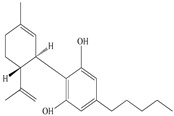	*D. morbifera*	Antioxidant, anti-inflammatory and neuroprotective
(−)-Bremazocine	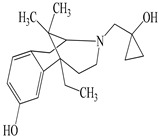	*D. morbifera*	κ-opioid agonist
Doxapram	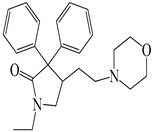	*D. morbifera*	Respiratory stimulant
Resolvin D2	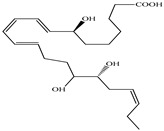	*D. morbifera*	Anti-inflammatory
Procyclidine	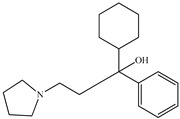	*D. morbifera*	Anticholinergic agent
2-arachidonoylglycerol	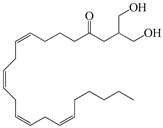	*D. morbifera*	Cannabimimetic activity
Eplerenone	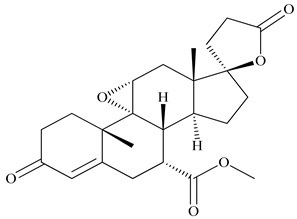	*D. morbifera*	Prevention of heart failure and mild symptoms
*Cis*-6-oxogeran-4-enyl-10-oxy-*O*-β-arabinopyranosyl -4′-*O*-β-arabinopyranosyl-2”-octadec-9”’,12”’,15”’-trienoate	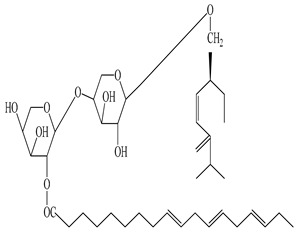	*D. morbifera*	Antioxidant
Geran-3(10)-enyl-1-oxy-*O*-β-arabinopyranosyl -4′-*O*-β-arabinopyranosyl-2”-octadec-9”’, 12”’,15”’-trienoate	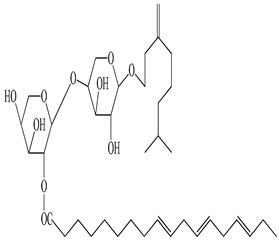	*D. morbifera*	Antioxidant
Geranilan-8-oxy-*O*-α-d-xylopyranosyl -20-n-octadec-9”,12”,15”-trienoate	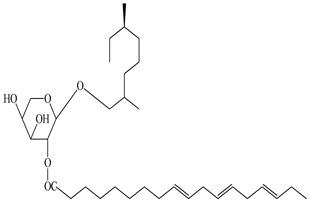	*D. morbifera*	Antioxidant
1-cyclohex-20,50-dienyl 1-cyclohexylethanol -*O*-β-D-xylopyranoside	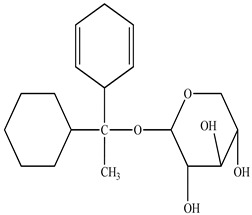	*D. morbifera*	Antioxidant
(9Z,16S)-16-Hydroxy-9,17-octadecadiene -12,14-diynoic acid	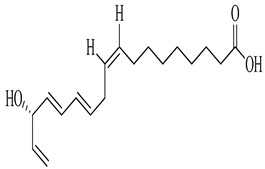	*D. morbifera*	Anti-complement activity
Dendropanoxide	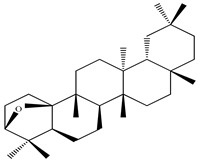	*D. morbifera*	Anti-complement activity and anti-osteoclastogenic activity
α-Glutinol	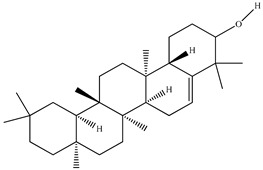	*D. morbifera*	Anti-complement activity
β-Amyrin	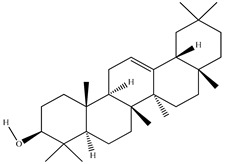	*D. morbifera*	Anti-complement activity and anti-osteoclastogenic activity
α-Amyrin	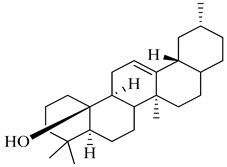	*D. morbifera*	Anti-complement activity
Trans-Phytol	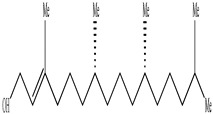	*D. morbifera*	Anti-complement activity
β-Sitosterol	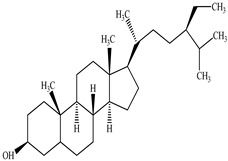	*D. morbifera*	Anti-complement activity
Friedelin	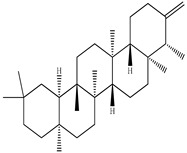	*D. morbifera*	Anti-osteoclastogenic activity
Epifriedelanol	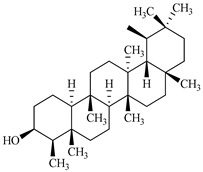	*D. morbifera*	Anti-osteoclastogenic activity
Gallic acid	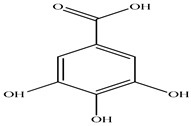	*D. morbifera*	Antioxidant, anti-inflammatory and anti-cancer activity
2,5-Dihydroxybenzoic acid	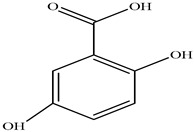	*D. morbifera*	Antioxidant, anti-inflammatory and anti-cancer activity
Catechin	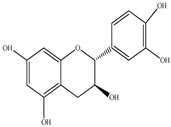	*D. morbifera*	Antioxidant, anti-inflammatory and anti-cancer activity
4-Hydroxybenzoic acid	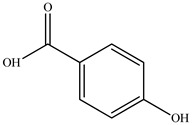	*D. morbifera*	Antioxidant, anti-inflammatory and anti-cancer activity
Caffeic acid	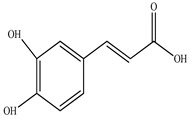	*D. morbifera*	Antioxidant, anti-inflammatory and anti-cancer activity
Syringic acid	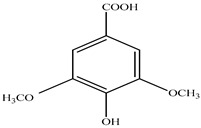	*D. morbifera*	Antioxidant, anti-inflammatory and anti-cancer activity
p-Coumaric acid	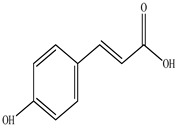	*D. morbifera*	Antioxidant, anti-inflammatory and anti-cancer activity
Trans-Ferulic acid	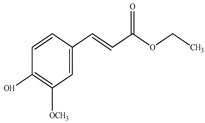	*D. morbifera*	Antioxidant, anti-inflammatory and anti-cancer activity
Salicylic acid	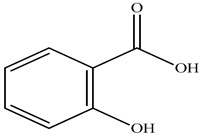	*D. morbifera*	Antioxidant, anti-inflammatory and anti-cancer activity
Hesperidin	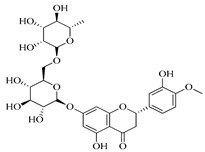	*D. morbifera*	Antioxidant, anti-inflammatory and anti-cancer activity
Naringin	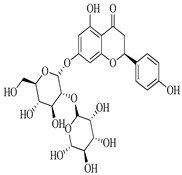	*D. morbifera*	Antioxidant, anti-inflammatory and anti-cancer activity
Resveratrol	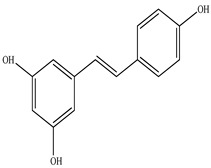	*D. morbifera*	Antioxidant, anti-inflammatory and anti-cancer activity
Myricetin	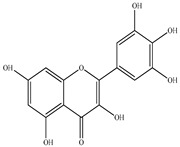	*D. morbifera*	Antioxidant, anti-inflammatory and anti-cancer activity
Trans-Cinnamic acid	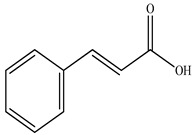	*D. morbifera*	Antioxidant, anti-inflammatory and anti-cancer activity

**Table 3:** Potential mechanisms of efficacy for some pharmacologic properties of *D. morbifera.*

Activity Tested	Extract	Plant Part	Model	Major Effects	Reference
Antioxidant	Ethanol	Boughs	DPPH-radical scavenging assays	*-D. morbifera* extracts showed similar antioxidant capacities (82.92 ± 0.49%) to that of the vitamin C (90.11 ± 0.13%) positive control at the same concentration of 500 µg/mL	[35]
Antioxidant	Fermented	Leaves	DPPH-radical scavenging assays	-Exhibited activities with IC_50_ value of 65.30 µg/mL	[16]
Antioxidant	Methanol	Leaves, debarked stems, and bark	DPPH-radical scavenging assays	-Exhibited activities with RC_50_ values of 16.7 µg/mL for debarked stem extracts, 31.6 µg/mL for yellow leaves extract, and 37.8 µg/mL green leaf extracts	[21]
Antioxidant	Hot water and ethanol	Leaves	ABTS-radical scavenging assays	-Exhibited activities with IC_50_ value of 3.79 mg/mL for hot water, 3.75 mg/mL for 30% ethanol, and 3.58 mg/mL for 60% ethanol	[25]
Antioxidant	Fermented	Leaves	Hydroxyl-radical scavenging activity	-Exhibited activities with IC_50_ value of 57.52 µg/mL	[50]
Antioxidant	Water	Stem and leaves	Sprague–Dawley rats	-*D. morbifera* extracts significant enhancements in GSH, SOD, and CAT activities and a reduction in the MDA level	[30]
Antioxidant	Water	Aerial part	Sprague–Dawley rats	-*D. morbifera* extracts significantly decreased the hepatic MDA content and ameliorated the SOD and GSH content.	[52]
Anti-inflammatory	Aqueous	Leaves	BV-2 cells	-*D. morbifera* extracts effectively attenuates NO production, cell viability, and proinflammatory mediators, and subsequently suppressed the phosphorylation of both the IκB-α and NF-κB p65 subunit and MAPK signaling	[13]
Anti-inflammatory	Ethanol	Leaves	RAW264.7 macrophages	-Doses of 200 and 400 µg/mL effectively inhibited the activity of inflammatory mediators NO, TNF-α, and IL-6	[17]
Anti-inflammatory	Ethyl acetate	Leaves	BV-2 cells	-*D. morbifera* treatment significantly attenuated the activation of MAPKs and NF-κB, and subsequently upregulated M2 polarization of alternative anti-inflammatory markers	[20]
Anti-inflammatory	Methanol	Leaves	RAW264.7 macrophages	-*D. morbifera* extracts significantly and dose-dependently reduced the production of NO and PGE2 and significantly inhibited protein and mRNA expression in COX-2 and iNOS activities and could modulate NF-κB and MAPK signaling -*D. morbifera* extracts significantly induced Nrf2 nuclear translocation and thereby induced HO-1 expression	[26]
Anti-inflammatory	Methanol	Leaves	Ear edema mouse model	Treatment with-*D. morbifera* extracts triggered a protective effect against the increase in ear thickness induced by TPA Treatment with-*D. morbifera* reversed ear edema and epidermal hyperproliferation, and increased the number of neutrophils induced by TPA	[26]
Anti-inflammatory	Fermented	Leaves	BALB/C mice	-*D. morbifera* extracts at 125, 250, and 500 mg/kg reduced levels of TNF-α, IL-2, IL-4, IL-5, IL-6, IL-10, IL-12, IL-12p70, IL-13 and IFN-γ in immunized BALB/C mice	[27]
Anti-inflammatory	Water	Stem and leaves	Sprague–Dawley rats	-*D. morbifera* extracts administered at 25 mg/kg markedly inhibited inflammatory cytokines and TGF-β1 expression in diabetic rats	[30]
Anti-inflammatory	Water	Aerial parts	Sprague–Dawley rats	- *D. morbifera* extracts at 25 mg/kg showed significant anti-inflammatory effects by reducing the levels of inflammatory cytokines such as TNF-α, IL-1β, and IL-6, and reversed IL-10 levels in a cisplatin-induced rat model	[62]
Anti-amnesic	Ethanol	Leaves	PC12 and MC-ⅨC cells	-*D. morbifera* extracts treatment effectively inhibited the AChE as an ACh-hydrolyzing enzyme in high glucose-induced PC12 and MC-ⅨC cells	[14]
Neuroprotective	Ethanol	Leaves	SH-SY5Y cells	-Rutin, a bioflavonoid isolated from *D. morbifera*, protected the higher level of intracellular Ca^2+^ and depleted the level of MMP, as well as subsequently decreased rotenone-induced ROS generation -Rutin prevented a decrease the levels of Bax/Bcl-2 ratio, caspase-9, and caspase-3	[63]
Neuroprotective	Ethyl acetate	Leaves	HT22 cells	-*D. morbifera* extracts significantly inhibited mitochondrial dysfunction and the elevation of Ca^2+^ levels and reversed subsequent AIF nuclear translocation in glutamate-induced HT22 mouse hippocampal neuronal cells	[64]
Neuroprotective	Ethanol	Stem	Sprague–Dawley rats	*-D. morbifera* administration ameliorates cognitive dysfunction via an increase in cell proliferation, neuroblast differentiation, and AChE activity in the hippocampus induced by cadmium-induced neurotoxicity	[14]
Neuroprotective	Water	Leaves	Sprague–Dawley rats	*-D. morbifera* extracts significantly reduced mercury levels in the hippocampus and ROS generation and reversed hippocampal activities in dimethylmercury-induced rats	[24]
Neuroprotective	Ethanol	Stems	Sprague–Dawley rats	*-D. morbifera* administration changes serum triiodothyronine (T3), thyroxine (T4), and thyroid-stimulating hormone levels in the hippocampus induced by hypothyroidism neurotoxicity	[28]
Neuroprotective	Ethanol	Leaves	C57BL/6 mice	*-D. morbifera* administration significantly improved D-galactose-induced reduction in microglial activation, escape latency, swimming speed, and spatial preference behavior	[65]
Neuroprotective	Aqueous	Leaves	C57BL/6 mice	*-D. morbifera* treatment effectively improved behavioral function, and protected dopaminergic neuronal loss by restoring TH levels in the brain tissue of MPTP-induced PD mice	[13]
Neuroprotective	Ethanol	Leaves	C57BL/6 mice	-Ethanol extracts of *D. morbifera* significantly improved glucose tolerance status, and behavioral impairments, and significantly protects the abnormal activity of mitochondria by inhibiting phosphorylated p-JNK, p-IRS, p-Akt, and p-tau in high-fat diet-induced mice	[66]
Anti-cancer	Methanol	Leaves and debarked stems	Huh-7 cells	*-D. morbifera* extracts showed strong induction of p53, and p16 inhibited the activation of ERK and reduced Akt levels and the suppression of Huh-7 cell proliferation	[21]
Anti-cancer	Silver nanoparticles	Leaves	A549 and HepG2	-Silver nanoparticles synthesized from *D. morbifera* enhanced ROS production in both cell lines and the modification of EGFR/p38 MAPK signaling	[67]
Anti-cancer	Ethanol	Stem bark	U937 cells	*-D. morbifera*-induced apoptosis in U937 cells was associated with the activation of caspase-8, -9, and -3 and downregulation of anti-apoptotic IAP family proteins	[68]
Anti-cancer	Water	Aerial parts	Sprague–Dawley rats	*-D. morbifera* administration protects against kidney damage induced by CDDP in tumor models	[62]
Anti-diabetes	Water	Leaves	3T3-L1 cells	*-D. morbiferus* treatment reduced intracellular triglyceride levels and glucose uptake by lowering protein and mRNA expression levels of adipogenesis-related genes	[9]
Anti-diabetes	Water	Leaves and stem	Sprague–Dawley rats	-*D. morbifera* administration protected body and organ weight loss, significantly increased BUN, and significantly reduced KIM-1, SBP1, and PKM2 levels in the urinary excretions of diabetic rats	[30]
Anti-diabetes	Methanol	Leaves	Sprague–Dawley rats	*-D. morbifera* administration showed significant hypoglycemic activity by decreasing the total cholesterol, serum glucose, urea, triglycerides, creatinine, uric acid, alanine aminotransferase (ALT), and aspartate aminotransferase (AST) levels in streptozotocin-induced diabetic rats	[22]
Anti-diabetes	Ethanol	Leaves	C57BL/6	-*D. morbifera* treatment protected against high-fat diet-induced abnormal mitochondrial activity and improved p-JNK, p-IRS, p-Akt, and p-tau in high-fat diet-induced diabetic mice	[14]
Anti-diabetes	Water and ethanol	Leaves and stem	ICR mice	*-D. morbifera* administration maintained a high level of body weight and increased insulin secretion by reducing the glucose concentration in the blood in streptozotocin-induced diabetic mice	[31]
Hepatoprotective	Ethanol	Root, leaves and stem	HepG2 cells	*-D. morbifera* exhibited strong antioxidant activity, and showed hepatoprotective activity against t-butyl hydroperoxide-induced HepG2 cells	[32]
Hepatoprotective	Aqueous	Leaves	Sprague–Dawley rats	*-D. morbifera* administration prevented ethanol-induced hepatotoxicity due to reductions of serum aspartate aminotransferase and alanine aminotransferase levels, and maintained enzymatic oxidant status, and suppressed cytochrome P-450 2E1 expression	[33]
Immunomodulatory	Fermented	Leaves	BALB/C mice	*-D. morbifera* administration showed an increase in spleen cells and CD8a+, CD11b, and CD3+ T-cell expression, and downregulated the IgG super-family	[27]
Immunomodulatory	Ethanol	Leaves, branch, sapling, and mixed	BALB/c mice	*-D. morbifera* administration significantly increased splenocyte cytokines, NO production, and LDH, and enhances innate immunity by modulator NF-κB signaling	[34]
Antimicrobial	Ethanol	Leaves	Paper disc test	With-*D. morbifera* extract concentrations of 40, 80, and 100 µg/mL, a 3.0 mm suggesting higher antibiotic effects against *S. mutans* and *C. albicans*	[35]
Antiplasmodial	Methanol	Stem bark	Semi-automated micro-dilution assay	-Extracts exhibited activities with IC_50_ values of 6.2 µg/mL and 5.3 µg/mL, against D10	[36]
Anticomplementary	Aqueous	Leaves	Classical and alternative pathway assay	-*D. morbifera* exhibited significant inhibitory activity against complementary system with IC_50_ values of 87.3 mM for (3S)-falcarinol, 15.2 mM for (3S,8S)-falcarindiol, and 39.8 mM for (3S)-diynene.	[53]
Anticomplementary	Methanol	Leaves	Classical pathway assay	-(9Z,16S)-16-hydroxy-9,17-octadecadiene-12,14-diynoic acid from *D. morbifera* exhibited activities with an IC_50_ value of 56.98 µM.	[47]
Cytotoxicity	Methanol	Leaves	MTT assay	-Extracts exhibited cytotoxic activities greater than 93% at doses of more than 100 μg/mL, and 6 to 11% at doses of less than 50 μg/mL	[35]
Cytotoxicity	Silver nanoparticles	Leaves	MTT assay	-D-AgNPs at 100 µg/mL showed potent cytotoxicity after 48 hours	[38]
Cytotoxicity	Methanol	Leaves	MTT assay	-Extracts exhibited low cytotoxicity with IC_50_ values exceeding 50 µg/mL, maintaining up to 80% cell viability induced by glutamate toxicity	[64]
Cytotoxicity	Aqueous	Leaves	MTT assay	-Extracts with a high concentration of *D. morbifera* of 500 μg/mL showed no toxic effects and maintained up to 100% cell viability induced by LPS toxicity	[13]
Toxicity	Water	Leaves	Sprague–Dawley rats	-No deaths were observed at the highest concentration tested, and the LD50 values for both extracts was above 2000 mg/kg body weight	[37]

## Supplementary Materials

The following are available online at https://www.mdpi.com/2076-3921/9/10/962/s1, Figure S1: The total number of *Dendropanax* genus-related publications registered in Pub Med literature database. Tables S1 and S2: In recent decades, the phytochemical and main bioactive compounds from different species of the *Dendropanax* genus have been studied.

## Abbreviations

WHO: World health organization; ROS, Reactive oxygen species; SOD, Superoxide dismutase; GPx, Glutathione peroxidase; CAT, Catalase; DPPH, 2, 2-dipheny-l-picrylhydrazyl; ABTS, 2, 2’-azinobis-3-ethylbenzothiazoline-6-sulphonate; MDA, Malondialdehyde; GSH, Glutathione; GST, Glutathione-S-transferase; SOD1, Zn-superoxide dismutase; NF-κB, Nuclear factor kappa B; LPS, Lipopolysaccharide; NO, Nitric oxide; TNF-α, Tumor necrosis factor-alpha; IL-6, interleukin-6; PGE_2_, Prostaglandin E_2_; IL-1β, interleukin-1 β; iNOS, Inducible nitric oxide synthase; COX-2, Cyclooxygenase-2; AChE, Acetylcholinesterase; Ach Acetylcholine; VEGF, Vascular endothelial growth factor; CREB, cAMP response element binding protein; BDNF, Brain-derived neurotrophic factor; MAPK, Mitogen-activated protein kinase; MMP, Mitochondrial membrane potential; TH, Tyrosine hydroxylase; MTT, 3-(4,5-dimethylthiazol-2-yl)-2,5-diphenyltetrazolium bromide; COLO-205, Colon adenocarcinoma cells; HOS, Clonal human osteosarcoma cells; SNU-245 and SNU-308, Biliary tract cells; Huh-BAT and Huh-7, Hepatocellular carcinoma cells; OS, Human osteosarcoma; ERK, Extracellular receptor kinase; AKT, Reduced protein kinase B; MAP1LC3, Microtubule-associated protein 1 light chain 3; Nrf2, Phospho-nuclear factor erythroid 2-related factor; EndoG, Endonuclease G; BUN, Blood urea nitrogen; KIM-1, Kidney injury molecule-1; SBP1, Selenium binding protein-1; PKM2, Pyruvate kinase muscle isozyme M2; ALT, Alanine amino transferase; AST, Aspartate amino transferase; LDH, Lactate dehydrogenase; MPTP, 1-methyl-4-phenyl-1, 2, 3, 6-tetrahydropyridine; UPLC-QTOF/MS, Ultra-performance liquid chromatography/quadrupole time of flight tandem mass spectrometry; N/A, Not applicable.
