# Supplementary material for: Dendropanax Morbiferus and Other Species from the Genus Dendropanax: Therapeutic Potential of Its Traditional Uses, Phytochemistry, and Pharmacology
Source: Antioxidants (Basel). 2020 Oct 8;9(10):962. doi: 10.3390/antiox9100962 (PMC7601828; doi:10.3390/antiox9100962)
Supplement: Supplementary file 1 [file antioxidants-09-00962-s001.pdf]

antioxidants

MDPI

Supplementary material

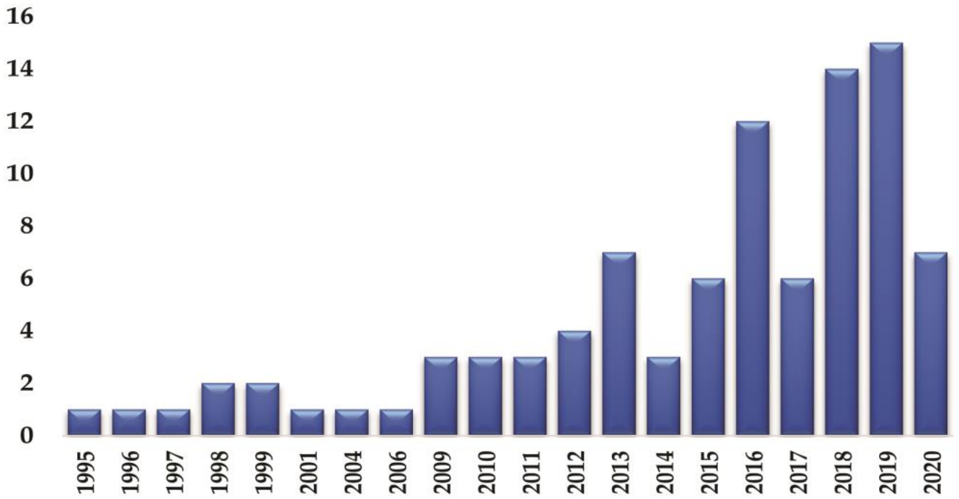

Figure S1. The total number of *Dendropanax* genus-related publications registered in Pub Med literature database**.** The y-axis shows the crude total numbers of full-length articles in PubMed. The x-axis shows the year articles first appeared on PubMed.

Table S1. Phytochemical constituents identified from *Dendropanax* species

Serial. No	Compound	Molecular Formula	Plant	Plant Part	Extraction method	Content in the extract	References
			D. arboreus				
1	Falcarinol	$C_{17}H_{22}O$	*D. arboreus*	Leaves	Methanol	500 mg/488g	[1]
2	Cis-1,9,16-heptadecatriene-4,6-diyne-3,8-diol	$C_{17}H_{22}O_2$	*D. arboreus*	Leaves	80% Ethanol	112 mg/322g	[2]
3	Dehydrofalcarindiol	$C_{17}H_{22}O_2$	*D. arboreus*	Leaves	Methanol	43 mg/488g	[1]
4	Dehydrofalcarinol	$C_{17}H_{22}O$	*D. arboreus*	Leaves	Methanol	30 mg/488g	[1]
5	Falcarindiol	$C_{17}H_{24}O_2$	*D. arboreus*	Leaves	Methanol	26 mg/488g	[1]
6	(3S)-Diynene	$C_{17}H_{22}O$	*D. arboreus*	Leaves	Methanol	21 mg/488g	[1]
7	(3S)-16,17-Didehydrofalcarinol	$C_{17}H_{22}O$	*D. arboreus*	Leaves	Methanol	7.4mg/488g	[1]
8	Dendroarboreols A	$C_{17}H_{22}O_2$	*D. arboreus*	Leaves	Methanol	1.8 mg/488g	[1]
9	Dendroarboreols B	$C_{17}H_{22}O_2$	*D. arboreus*	Leaves	Methanol	1.8 mg/488g	[1]
			(% of constituent of the essential oil (2 mg))				
10	valerianol	$C_{15}H_{26}O$	*D. arboreus*	Leaves	Essential oils	21.2 %	[3]
11	γ-eudesmol	$C_{15}H_{26}O$	*D. arboreus*	Leaves	Essential oils	16.3 %	[3]
12	Germacrene B	$C_{15}H_{24}$	*D. arboreus*	Leaves	Essential oils	8.8 %	[3]
13	14-hydroxy-α-muurolene	$C_{15}H_{24}O$	*D. arboreus*	Leaves	Essential oils	5.2 %	[3]
14	Khusinol	$C_{15}H_{24}O$	*D. arboreus*	Leaves	Essential oils	4.3 %	[3]
15	δ-Cadinene	$C_{15}H_{24}$	*D. arboreus*	Leaves	Essential oils	3.8 %	[3]
16	γ-Elemene	$C_{15}H_{24}$	*D. arboreus*	Leaves	Essential oils	1.8 %	[3]
17	Trans-β-guaiene	$C_{15}H_{24}$	*D. arboreus*	Leaves	Essential oils	1.4 %	[3]
18	Germacrene D	$C_{15}H_{24}$	*D. arboreus*	Leaves	Essential oils	1.3 %	[3]
19	Valencene	$C_{15}H_{24}$	*D. arboreus*	Leaves	Essential oils	1.1 %	[3]
21	γ-Muurolene	$C_{15}H_{24}$	*D. arboreus*	Leaves	Essential oils	1.1 %	[3]

22	β-Bourbonene	$C_{15}H_{24}$	*D. arboreus*	Leaves	Essential oils	0.7 %	[3]
23	(E)-2-decenal	$C_{10}H_{18}O$	*D. arboreus*	Leaves	Essential oils	0.4 %	[3]
24	β-Gurjunene	$C_{15}H_{24}$	*D. arboreus*	Leaves	Essential oils	0.4 %	[3]
25	Khusinol acetate	$C_{17}H_{26}O_2$	*D. arboreus*	Leaves	Essential oils	0.3 %	[3]
26	14-hydroxy-δ-cadinene	$C_{15}H_{24}O$	*D. arboreus*	Leaves	Essential oils	0.2 %	[3]
27	Octanol	$C_8H_{18}O$	*D. arboreus*	Leaves	Essential oils	0.2 %	[3]
28	*trans*-Verbenol	$C_{10}H_{16}O$	*D. arboreus*	Leaves	Essential oils	0.2 %	[3]
29	Myrtenol	$C_{10}H_{16}O$	*D. arboreus*	Leaves	Essential oils	0.2 %	[3]
30	Hexadecanoic acid	$C_{16}H_{32}O_2$	*D. arboreus*	Leaves	Essential oils	0.2 %	[3]
31	Isovaleraldehyde	$C_5H_{10}O$	*D. arboreus*	Leaves	Essential oils	<0.1 %	[3]
32	Methyl crotonate	$C_5H_8O_2$	*D. arboreus*	Leaves	Essential oils	<0.1 %	[3]
33	Isoamyl alcohol	$C_5H_{12}O$	*D. arboreus*	Leaves	Essential oils	<0.1 %	[3]
34	2-methyl-2-butenal	C_5H_8O	*D. arboreus*	Leaves	Essential oils	<0.1 %	[3]
35	Hexanal	$C_6H_{12}O$	*D. arboreus*	Leaves	Essential oils	<0.1 %	[3]
36	Furfural	$C_5H_4O_2$	*D. arboreus*	Leaves	Essential oils	<0.1 %	[3]
37	(Z)-2-Hexenal	$C_6H_{10}O$	*D. arboreus*	Leaves	Essential oils	<0.1 %	[3]
38	Hexanol	$C_6H_{14}O$	*D. arboreus*	Leaves	Essential oils	<0.1 %	[3]
39	2-Heptanone	$C_7H_{14}O$	*D. arboreus*	Leaves	Essential oils	<0.1 %	[3]
40	2-Heptanol	$C_7H_{16}O$	*D. arboreus*	Leaves	Essential oils	<0.1 %	[3]
41	Heptanal	$C_7H_{14}O$	*D. arboreus*	Leaves	Essential oils	<0.1 %	[3]
42	(E, E)-2,4-hexadienal	C_6H_8O	*D. arboreus*	Leaves	Essential oils	<0.1 %	[3]
43	α-Thujene	$C_{10}H_{16}$	*D. arboreus*	Leaves	Essential oils	<0.1 %	[3]
44	Camphene	$C_{10}H_{16}$	*D. arboreus*	Leaves	Essential oils	<0.1 %	[3]
45	Thuja-2,4(10)-diene	$C_{10}H_{14}$	*D. arboreus*	Leaves	Essential oils	<0.1 %	[3]
46	Benzaldehyde	C_7H_6O	*D. arboreus*	Leaves	Essential oils	<0.1 %	[3]
47	Heptanol	$C_7H_{16}O$	*D. arboreus*	Leaves	Essential oils	<0.1 %	[3]

48	Sabinene	$C_{10}H_{16}$	*D. arboreus*	Leaves	Essential oils	<0.1 %	[3]
49	6-methyl-5-hepten-2-one	$C_8H_{14}O$	*D. arboreus*	Leaves	Essential oils	<0.1 %	[3]
50	Octanal	$C_8H_{16}O$	*D. arboreus*	Leaves	Essential oils	<0.1 %	[3]
51	α-Phellandrene	$C_{10}H_{16}$	*D. arboreus*	Leaves	Essential oils	<0.1 %	[3]
52	(E, E)-2,4-Heptadienal	$C_7H_{10}O$	*D. arboreus*	Leaves	Essential oils	<0.1 %	[3]
53	α-Terpinene	$C_{10}H_{16}$	*D. arboreus*	Leaves	Essential oils	<0.1 %	[3]
54	p-Menth-1-ene	$C_{10}H_{18}$	*D. arboreus*	Leaves	Essential oils	<0.1 %	[3]
55	1,8-Cineole	$C_{10}H_{18}O$	*D. arboreus*	Leaves	Essential oils	<0.1 %	[3]
56	(Z)-β-ocimene	$C_{10}H_{16}$	*D. arboreus*	Leaves	Essential oils	<0.1 %	[3]
57	(E)-β-ocimene	$C_{10}H_{16}$	*D. arboreus*	Leaves	Essential oils	<0.1 %	[3]
58	(E)-2-octenal	$C_8H_{14}O$	*D. arboreus*	Leaves	Essential oils	<0.1 %	[3]
59	γ-Terpinene	$C_{10}H_{16}$	*D. arboreus*	Leaves	Essential oils	<0.1 %	[3]
60	Heptanoic acid	$C_7H_{14}O_2$	*D. arboreus*	Leaves	Essential oils	<0.1 %	[3]
61	2-nonanone	$C_9H_{18}O$	*D. arboreus*	Leaves	Essential oils	<0.1 %	[3]
62	Methyl benzoate	$C_8H_8O_2$	*D. arboreus*	Leaves	Essential oils	<0.1 %	[3]
63	Nonanal	$C_9H_{18}O$	*D. arboreus*	Leaves	Essential oils	<0.1 %	[3]
64	Isophorone	$C_9H_{14}O$	*D. arboreus*	Leaves	Essential oils	<0.1 %	[3]
65	α-Campholenal	$C_{10}H_{16}O$	*D. arboreus*	Leaves	Essential oils	<0.1 %	[3]
66	*cis*-Limonene oxide	$C_{10}H_{16}O$	*D. arboreus*	Leaves	Essential oils	<0.1 %	[3]
67	*trans*-Sabinol	$C_{10}H_{16}O$	*D. arboreus*	Leaves	Essential oils	<0.1 %	[3]
68	(E)-2-Nonenal	$C_9H_{16}O$	*D. arboreus*	Leaves	Essential oils	<0.1 %	[3]
69	Isopinocamphone	$C_{10}H_{16}O$	*D. arboreus*	Leaves	Essential oils	<0.1 %	[3]
70	Terpinen-4-ol	$C_{10}H_{18}O$	*D. arboreus*	Leaves	Essential oils	<0.1 %	[3]
71	α-Terpineol	$C_{10}H_{18}O$	*D. arboreus*	Leaves	Essential oils	<0.1 %	[3]
72	Decanal	$C_{10}H_{20}O$	*D. arboreus*	Leaves	Essential oils	<0.1 %	[3]
73	*trans*-Piperitol	$C_{10}H_{18}O$	*D. arboreus*	Leaves	Essential oils	<0.1 %	[3]

74	Verbenone	$C_{10}H_{14}O$	*D. arboreus*	Leaves	Essential oils	<0.1 %	[3]
75	*trans*-Carveol	$C_{10}H_{16}O$	*D. arboreus*	Leaves	Essential oils	<0.1 %	[3]
76	Nerol	$C_{10}H_{18}O$	*D. arboreus*	Leaves	Essential oils	<0.1 %	[3]
77	Cuminaldehyde	$C_{10}H_{12}O$	*D. arboreus*	Leaves	Essential oils	<0.1 %	[3]
78	Carvone	$C_{10}H_{14}O$	*D. arboreus*	Leaves	Essential oils	<0.1 %	[3]
79	Decanol	$C_{10}H_{22}O$	*D. arboreus*	Leaves	Essential oils	<0.1 %	[3]
80	(E)-anethole	$C_{10}H_{12}O$	*D. arboreus*	Leaves	Essential oils	<0.1 %	[3]
81	Bornyl acetate	$C_{12}H_{20}O_2$	*D. arboreus*	Leaves	Essential oils	<0.1 %	[3]
82	Safrole	$C_{10}H_{10}O_2$	*D. arboreus*	Leaves	Essential oils	<0.1 %	[3]
83	Thymol	$C_{10}H_{14}O$	*D. arboreus*	Leaves	Essential oils	<0.1 %	[3]
84	(E, Z)-2,4-decadienal	$C_{10}H_{16}O$	*D. arboreus*	Leaves	Essential oils	<0.1 %	[3]
85	δ-Elemene	$C_{15}H_{24}$	*D. arboreus*	Leaves	Essential oils	<0.1 %	[3]
86	α-Cubebene	$C_{15}H_{24}$	*D. arboreus*	Leaves	Essential oils	<0.1 %	[3]
87	α-Ylangene	$C_{15}H_{24}$	*D. arboreus*	Leaves	Essential oils	<0.1 %	[3]
88	α-Copaene	$C_{15}H_{24}$	*D. arboreus*	Leaves	Essential oils	<0.1 %	[3]
89	β-Cubebene	$C_{15}H_{24}$	*D. arboreus*	Leaves	Essential oils	<0.1 %	[3]
90	β-Elemene	$C_{15}H_{24}$	*D. arboreus*	Leaves	Essential oils	<0.1 %	[3]
91	*trans*-β-guaiene	$C_{15}H_{24}$	*D. arboreus*	Leaves	Essential oils	<0.1 %	[3]
92	γ-Cadinene	$C_{15}H_{24}$	*D. arboreus*	Leaves	Essential oils	<0.1 %	[3]
93	(E)-Phytyl acetate	$C_{22}H_{42}O_2$	*D. arboreus*	Leaves	Essential oils	<0.1 %	[3]
94	Dehydrofalcarindiol-A	$C_{17}H_{22}O_2$	*D. arboreus*	Leaves	Essential oils	<0.1 %	[3]
95	Dehydrofalcarindiol-B	$C_{17}H_{22}O_2$	*D. arboreus*	Leaves	Essential oils	<0.1 %	[3]
			D. dentiger				
96	Kaempferol 3-*O*-rutinoside	$C_{27}H_{30}O_{15}$	*D. dentiger*	Leaves	95% Ethanol	99 mg/207g	[4]
97	Sargentol	$C_{17}H_{24}O_{10}$	*D. dentiger*	Leaves	95% Ethanol	51.8 mg/207g	[4]
98	Kelampayoside A	$C_{20}H_{30}O_{13}$	*D. dentiger*	Leaves	95% Ethanol	28.9 mg/207g	[4]

99	Icariside F2	$C_{18}H_{26}O_{10}$	*D. dentiger*	Leaves	95% Ethanol	25.4 mg/207g	[4]
100	Quercetin 3-*O*-robinobioside	$C_{27}H_{30}O_{16}$	*D. dentiger*	Leaves	95% Ethanol	13.6 mg/207g	[4]
101	Dhurrin	$C_{14}H_{17}NO_7$	*D. dentiger*	Leaves	95% Ethanol	8.4 mg/207g	[4]
102	Coniferin	$C_{16}H_{22}O_8$	*D. dentiger*	Leaves	95% Ethanol	5.9 mg/207g	[4]
103	6'-*O*-Apiofuranosyl dendranthemoside A	$C_{24}H_{41}O_{12}$	*D. dentiger*	Leaves	95% Ethanol	4.6 mg/207g	[4]
104	β-D-glucopyranoside	$C_7H_{14}O_6$	*D. dentiger*	Leaves	95% Ethanol	4.3 mg/207g	[4]
105	Glucosyringic acid	$C_{15}H_{20}O_{10}$	*D. dentiger*	Leaves	95% Ethanol	1.6 mg/207g	[4]
106	2-(β-D-Glucopyranosyl)-3-isoxazolin-5-one	$C_9H_{13}NO_7Na$	*D. dentiger*	Leaves	95% Ethanol	1.2 mg/207g	[4]
107	3-Methoxy-D-mannono-1,4-lactone	$C_7H_{12}O_6Na$	*D. dentiger*	Leaves	95% Ethanol	1.2 mg/207g	[4]
108	Friedelan-3-one	$C_{30}H_{50}O$	*D. dentiger*	Stems	80% Ethanol	N/A	[5]
109	Glycerol monooleate	$C_{21}H_{42}O_4$	*D. dentiger*	Stems	80% Ethanol	N/A	[5]
110	Behenic acid	$C_{22}H_{44}O_2$	*D. dentiger*	Stems	80% Ethanol	N/A	[5]
111	1-Triacontanol	$C_{30}H_{62}O$	*D. dentiger*	Stems	80% Ethanol	N/A	[5]
112	Stearic acid	$C_{18}H_{36}O_2$	*D. dentiger*	Stems	80% Ethanol	N/A	[5]
113	Syringaldehyde	$C_9H_{10}O_4$	*D. dentiger*	Stems	80% Ethanol	N/A	[5]
114	Coniferyl aldehyde	$C_{10}H_{10}O_3$	*D. dentiger*	Stems	80% Ethanol	N/A	[5]
115	Daucosterol	$C_{35}H_{60}O_6$	*D. dentiger*	Stems	80% Ethanol	N/A	[5]
116	Sinapaldehyde	$C_{11}H_{12}O_4$	*D. dentiger*	Stems	80% Ethanol	N/A	[5]
117	Luteolin	$C_{15}H_{10}O_6$	*D. dentiger*	Stems	80% Ethanol	N/A	[5]
118	Sinapaldehyde glucoside	$C_{17}H_{22}O_9$	*D. dentiger*	Stems	80% Ethanol	N/A	[5]
119	(E)-4-Hydroxycinnamic acid	$C_9H_8O_3$	*D. dentiger*	Stems	80% Ethanol	N/A	[5]
120	(*E*)-Cinnamic acid	$C_9H_8O_2$	*D. dentiger*	Stems	80% Ethanol	N/A	[5]
121	(+)-syringaresinol-di-*O*-β-D-glucopyranoside	$C_{34}H_{46}O_{18}$	*D. dentiger*	Stems	80% Ethanol	N/A	[5]
122	Eugenol rutinoside	$C_{22}H_{32}O_{11}$	*D. dentiger*	Stems	80% Ethanol	N/A	[5]
123	Icariside E5	$C_{26}H_{34}O_{11}$	*D. dentiger*	Stems	80% Ethanol	N/A	[5]
D. gonatopodus (% of constituent of the essential oil (5.2 mg))							

124	(*E*)-2-Hexenal	$C_6H_{10}O$	*D. gonatopodus*	leaves	Essential oils	16.9 %	[6]
125	Terpinolene	$C_{10}H_{16}$	*D. gonatopodus*	leaves	Essential oils	14.8 %	[6]
126	δ-Cadinene	$C_{15}H_{24}$	*D. gonatopodus*	leaves	Essential oils	13.5 %	[6]
127	(*E*)-Caryophyllene	$C_{15}H_{24}$	*D. gonatopodus*	leaves	Essential oils	9.9 %	[6]
128	α-Copaene	$C_{15}H_{24}$	*D. gonatopodus*	leaves	Essential oils	7.3 %	[6]
129	*p*-Cymen-8-ol	$C_{10}H_{14}O$	*D. gonatopodus*	leaves	Essential oils	5.2 %	[6]
130	(*E*, *E*)-α-Farnesene	$C_{15}H_{24}$	*D. gonatopodus*	leaves	Essential oils	4.5 %	[6]
131	Germacrene-D	$C_{15}H_{24}$	*D. gonatopodus*	leaves	Essential oils	3.9 %	[6]
132	α-Pinene	$C_{10}H_{16}$	*D. gonatopodus*	leaves	Essential oils	3.9 %	[6]
133	α-Humulene	$C_{15}H_{24}$	*D. gonatopodus*	leaves	Essential oils	3.0 %	[6]
134	Myrcene	$C_{10}H_{16}$	*D. gonatopodus*	leaves	Essential oils	3.0 %	[6]
135	β-Pinene	$C_{10}H_{16}$	*D. gonatopodus*	leaves	Essential oils	2.8 %	[6]
136	Bicyclogermacrene	$C_{15}H_{24}$	*D. gonatopodus*	leaves	Essential oils	2.5 %	[6]
137	*p*-Cymene	$C_{10}H_{14}$	*D. gonatopodus*	leaves	Essential oils	1.9 %	[6]
138	Limonene	$C_{10}H_{16}$	*D. gonatopodus*	leaves	Essential oils	1.8 %	[6]
139	γ-Cadinene	$C_{15}H_{24}$	*D. gonatopodus*	leaves	Essential oils	0.7 %	[6]
140	Spathulenol	$C_{15}H_{24}O$	*D. gonatopodus*	leaves	Essential oils	0.6 %	[6]
141	Globulol	$C_{15}H_{26}O$	*D. gonatopodus*	leaves	Essential oils	0.6 %	[6]
142	α-Cadinol	$C_{15}H_{26}O$	*D. gonatopodus*	leaves	Essential oils	0.6 %	[6]
143	τ-Cadinol	$C_{15}H_{26}O$	*D. gonatopodus*	leaves	Essential oils	0.5 %	[6]
144	Mint sulfide	$C_{15}H_{24}S$	*D. gonatopodus*	leaves	Essential oils	0.4 %	[6]
145	α-Calacorene	$C_{15}H_{24}$	*D. gonatopodus*	leaves	Essential oils	0.4 %	[6]
146	γ-Muurolene	$C_{15}H_{24}$	*D. gonatopodus*	leaves	Essential oils	0.4 %	[6]
147	Aromadendrene	$C_{15}H_{24}$	*D. gonatopodus*	leaves	Essential oils	0.3 %	[6]
148	Alloaromadendrene	$C_{15}H_{24}$	*D. gonatopodus*	leaves	Essential oils	0.3 %	[6]
149	(*E*)-β-Ocimene	$C_{10}H_{16}$	*D. gonatopodus*	leaves	Essential oils	0.2 %	[6]

150	Linalool	$C_{10}H_{18}O$	*D. gonatopodus*	leaves	Essential oils	trace	[6]
151	β-Cubebene	$C_{15}H_{24}$	*D. gonatopodus*	leaves	Essential oils	trace	[6]
152	β-Elemene	$C_{15}H_{24}$	*D. gonatopodus*	leaves	Essential oils	trace	[6]
153	γ-Gurjunene	$C_{15}H_{24}$	*D. gonatopodus*	leaves	Essential oils	trace	[6]
154	(*E*)-β-Ionone	$C_{13}H_{20}O$	*D. gonatopodus*	leaves	Essential oils	trace	[6]
155	*trans*-Muurola-4(14),5-diene	$C_{15}H_{24}$	*D. gonatopodus*	leaves	Essential oils	trace	[6]
160	Ledene viridiflorene	$C_{15}H_{24}$	*D. gonatopodus*	leaves	Essential oils	trace	[6]
161	β-Calacorene	$C_{15}H_{20}$	*D. gonatopodus*	leaves	Essential oils	trace	[6]
162	Isoledene	$C_{15}H_{24}$	*D. gonatopodus*	leaves	Essential oils	trace	[6]
163	α-Gurjunene	$C_{15}H_{24}$	*D. gonatopodus*	leaves	Essential oils	trace	[6]
164	Viridiflorene	$C_{15}H_{24}$	*D. gonatopodus*	leaves	Essential oils	trace	[6]
165	Epiglobulol	$C_{15}H_{26}O$	*D. gonatopodus*	leaves	Essential oils	trace	[6]
166	Ledol	$C_{15}H_{26}O$	*D. gonatopodus*	leaves	Essential oils	trace	[6]
167	Viridiflorol	$C_{15}H_{26}O$	*D. gonatopodus*	leaves	Essential oils	trace	[6]
168	Palustrol	$C_{15}H_{26}O$	*D. gonatopodus*	leaves	Essential oils	trace	[6]
			D. capillaris (% of constituent of the essential oil (12 mg))				
169	6-3-Carene	$C_{10}H_{16}$	*D. capillaris*	leaves	Essential oils	44.7 %	[7]
170	Daucene	$C_{15}H_{24}$	*D. capillaris*	leaves	Essential oils	17.1 %	[7]
171	Dauca-5,8-diene	$C_{15}H_{24}$	*D. capillaris*	leaves	Essential oils	12.9 %	[7]
			D. Chevalieri				
172	Palmitic acid	$C_{16}H_{32}O_2$	*D. Chevalieri*	Roots	N/A	N/A	[8]
173	Dibutylphthalate	$C_{16}H_{22}O_4$	*D. Chevalieri*	Roots	N/A	N/A	[8]
174	Coniferaldehyde	$C_{10}H_{10}O_3$	*D. Chevalieri*	Roots	N/A	N/A	[8]
175	Scopoletin	$C_{10}H_8O_4$	*D. Chevalieri*	Roots	N/A	N/A	[8]
176	β-hydroxypropiovanillone	$C_{10}H_{12}O_4$	*D. Chevalieri*	Roots	N/A	N/A	[8]
177	(+)-Pinoresinol	$C_{20}H_{22}O_6$	*D. Chevalieri*	Roots	N/A	N/A	[8]

178	(+)-Syringaresinol	$C_{22}H_{26}O_8$	*D. Chevalieri*	Roots	N/A	N/A	[8]

Table S2. The main bioactive compounds of *Dendropanax* species and its pharmacological activities

Compound	Molecular structure	Plant species	Activity Tested
		D. gonatopodus	
β-Pinene	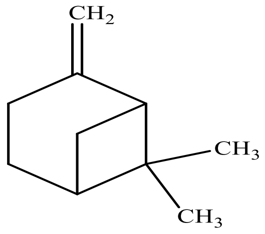	*D. gonatopodus*	Anti-oxidant and anti-inflammatory activity
p-Cymene	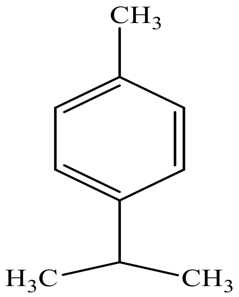	*D. gonatopodus*	Anti-oxidant and anti-inflammatory activity

Limonene	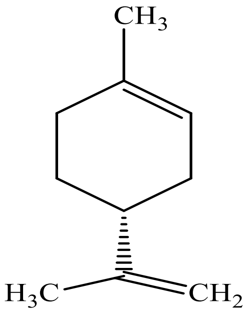	*D. gonatopodus*	Anti-oxidant and antibacterial activity
(*E*)-β-Ocimene	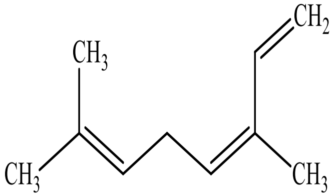	*D. gonatopodus*	Anti-oxidant and antibacterial activity
Terpinolene	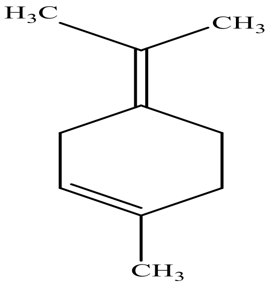	*D. gonatopodus*	Antioxidant, antiviral, and larvicidal Activity

Linalool	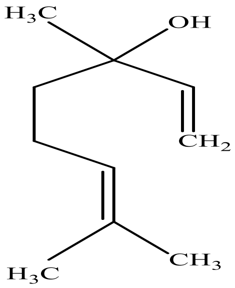	*D. gonatopodus*	Anti-oxidant and antimicrobial Activity
p-Cymen-8-ol	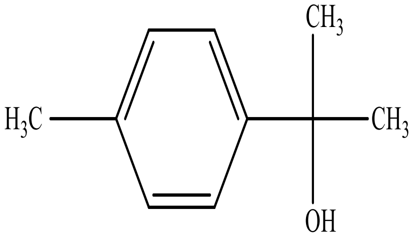	*D. gonatopodus*	Antioxidant and antifungal activity
α-Copaene	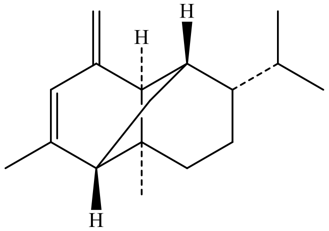	*D. gonatopodus*	Antioxidant activity
β-Cubebene	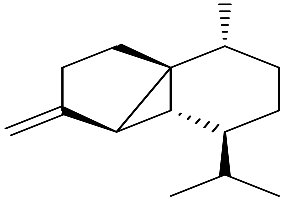	*D. gonatopodus*	Antioxidant, antimicrobial and larvicidal activity

β-Elemene	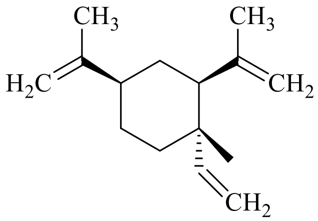	*D. gonatopodus*	Anti-oxidant, anticancer, cytotoxicity and anti-inflammatory activity
α-Humulene	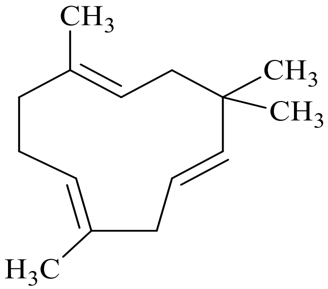	*D. gonatopodus*	Anti-Inflammatory, antioxidant, antibiotic, and cytotoxic activity
(E)-Caryophyllene	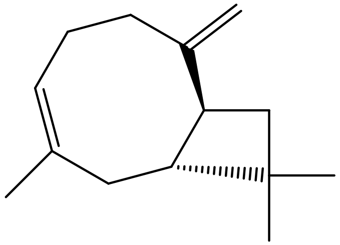	*D. gonatopodus*	Anticancer, antioxidant and antimicrobial activity
β-Copaene	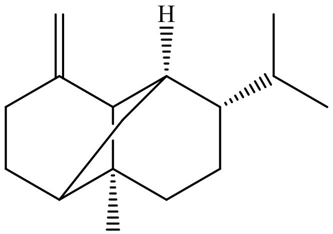	*D. gonatopodus*	Antioxidant and antibacterial activity

Aromadendrene	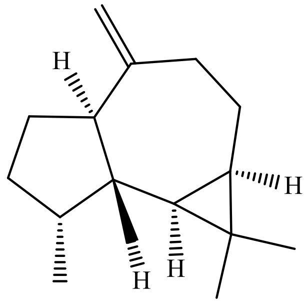	*D. gonatopodus*	Antibacterial and antioxidant activity
Alloaromadendrene	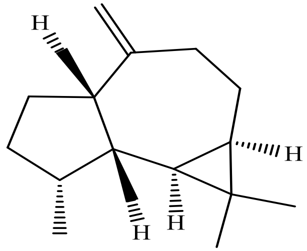	*D. gonatopodus*	Antineoplastic and antioxidant activity
Epiglobulol	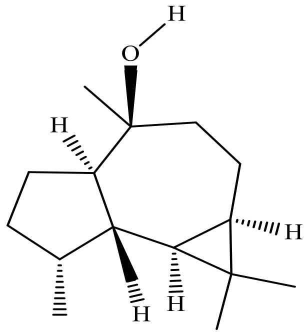	*D. gonatopodus*	Antibacterial and uterus relaxant activity

Viridiflorol	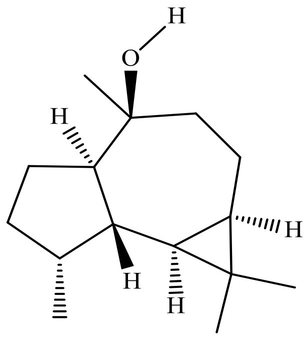	*D. gonatopodus*	Acetylcholinesterase inhibitory and antifungal activity
Globulol	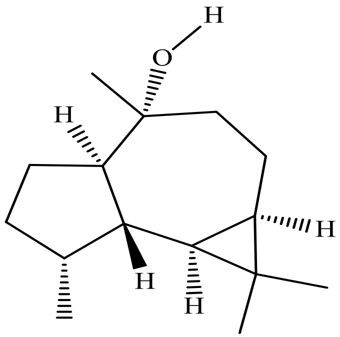	*D. gonatopodus*	Antibacterial, antifungal, antioxidant, sedative and anaesthetic activity
Spathulenol	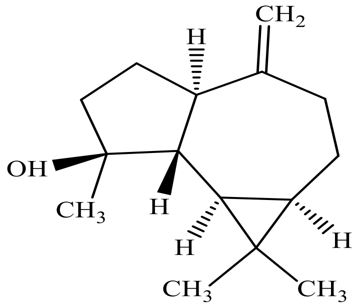	*D. gonatopodus*	Antibacterial, anticancer, anti-inflammatory, immunomodulatory and uterus relaxant activity

β-Ionone	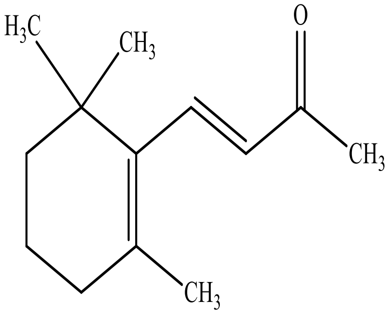	*D. gonatopodus*	Anticancer and antioxidant activity
γ- Muurolene	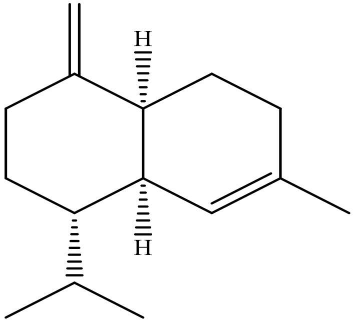	*D. gonatopodus*	Antimicrobial and antiseptic activity
γ-Gurjunene	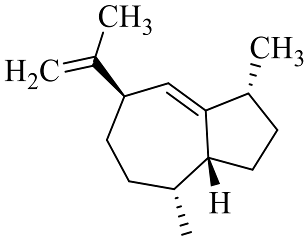	*D. gonatopodus*	Antimicrobial and antiseptic activity

δ-Cadinene	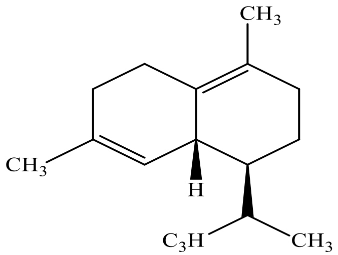	*D. gonatopodus*	Antimicrobial and antiseptic activity
α-Calacorene	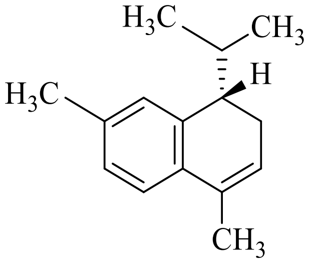	*D. gonatopodus*	Antimicrobial and antiseptic activity
γ-Cadinene	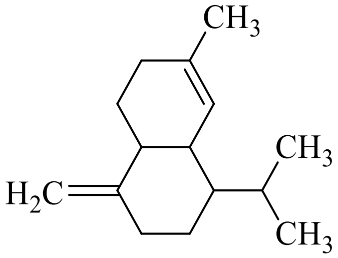	*D. gonatopodus*	Antibacterial activity
t-Cadinol	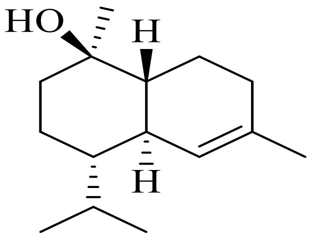	*D. gonatopodus*	Antibacterial activity

	D. capillaris		
α-Cadinol	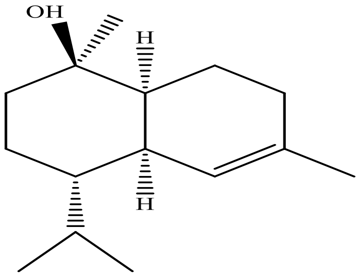	*D. capillaris*	Cytotoxicity
δ-3-Carene	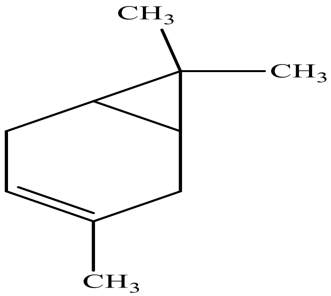	*D. capillaris*	Cytotoxicity
Dauca-5,8-diene	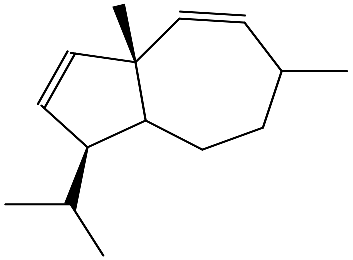	*D. capillaris*	Cytotoxicity
	D. Chevalieri		

Palmitic acid	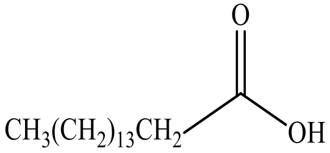	*D. Chevalieri*	Anti-inflammatory,
Dibutyl phthalate	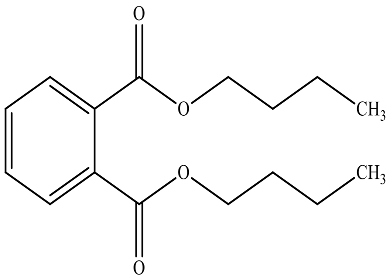	*D. Chevalieri*	Anti-inflammatory,
Scopoletin	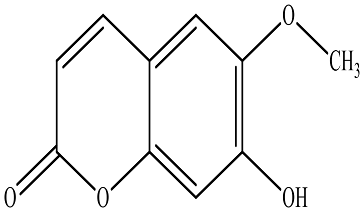	*D. Chevalieri*	Anti-inflammatory,
β-hydroxypropiovanillone	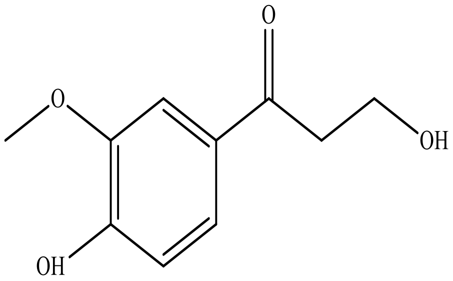	*D. Chevalieri*	Anti-inflammatory,

(+)-Pinoresinol	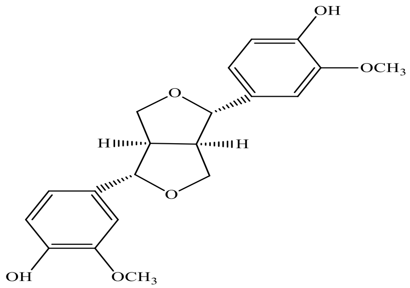	*D. Chevalieri*	Anti-inflammatory,
(+)-Syringaresinol	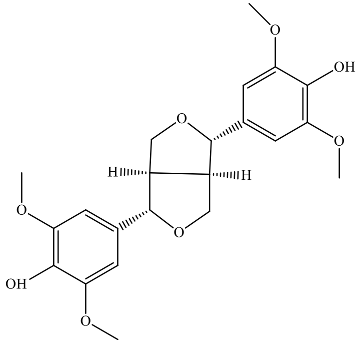	*D. Chevalieri*	Anti-inflammatory,
D. arboreus			
Falcarinol	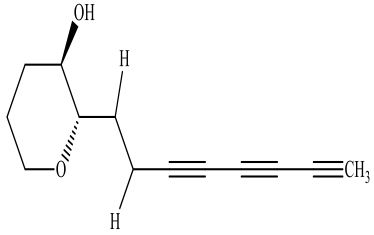	*D. arboreus*	Cytotoxicity

(3S)-16,17-didehydrofalcarinol	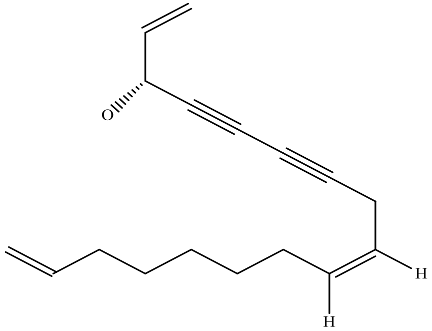	*D. arboreus*	Cytotoxicity
Dehydrofalcarinol-A	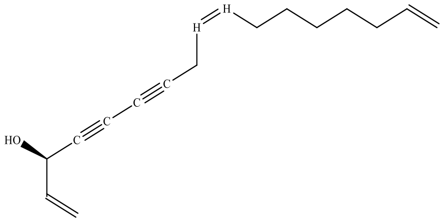	*D. arboreus*	Cytotoxicity
Dehydrofalcarindiol-B	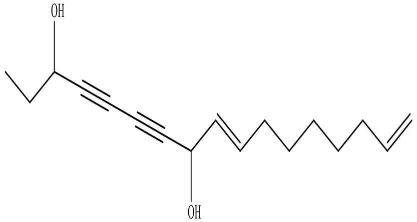	*D. arboreus*	Cytotoxicity

References

1. Bernart, M.W.; Cardellina, J.H.; Balaschak, M.S.; Alexander, M.R.; Shoemaker, R.H.; Boyd, M.R. Cytotoxic falcarinol oxylipins from *Dendropanax arboreus*. *J. Nat. Prod.* **1996**, *59*, 748–753.
2. Setzer, W.N.; Green, T.J.; Whitaker, K.W.; Moriarity, D.M.; Yancey, C.A.; Lawton, R.O.; Bates, R.B. A cytotoxic diacetylene from *Dendropanax arboreus*. *Planta Med.* **1995**, *61*, 470–471.
3. Pino, J.A.; Marbot, R.; Payo, A.; Chao, D.; Herrera, P.; Martí, M.P. Leaf oil of *dendropanax arboreus l.* from Cuba. *J. Essent. Oil Res.* **2005**, *17*, 547–548.
4. Lai, Y.C.; Lee, S.S. Chemical constituents from *Dendropanax dentiger*. *Nat. Prod. Commun.* **2013**, *8*, 1934578X1300800322.
5. Zheng, L.P.; He, Z.G.; Wu, Z.J.; Zhang, C. Chemical constituents from Dendropanax dentiger. *Chem. Nat. Compd.* **2012**, *48*, 883–885.
6. Setzer, W.N. Essential oil composition of *Dendropanax gonatopodus* from Monteverde, Costa Rica. An ab initio examination of aromadendrane sesquiterpenoids. *Nat. Prod. Commun.* **2008**, *3*, 1934578X0800300932.
7. Richmond, J.D.; Agius, B.R.; Wright, B.S.; Haber, W.A.; Moriarity, D.M.; Setzer, W.N. Essential oil compositions and cytotoxic activities of *Dendropanax capillaris*, *Oreopanax nubigenus*, and *Schefflera rodrigueziana* from Monteverde, Costa Rica. *Nat. Prod. Commun.* **2009**, *4*, 1934578X0900400221.
8. Ren, G.; Luo, Z.P.; Huang, H.L.; Shao, F.; Li, G.H.; Zhou, C.X.; Liu, R.H. [Study on the chemical constituents of the roots of *Dendropanax chevalieri*]. *Zhong Yao Cai* **2012**, *35*, 62–64.
